# Progressing towards Sustainable Machining of Steels: A Detailed Review

**DOI:** 10.3390/ma14185162

**Published:** 2021-09-08

**Authors:** Kashif Ishfaq, Irfan Anjum, Catalin Iulian Pruncu, Muhammad Amjad, M. Saravana Kumar, Muhammad Asad Maqsood

**Affiliations:** 1 Department of Industrial and Manufacturing Engineering, University of Engineering & Technology, Lahore 548900, Pakistan; irfanhanjum@gmail.com (I.A.); 2016im11@student.uet.edu.pk (M.A.M.); 2 Design, Manufacturing & Engineering Management, University of Strathclyde, Glasgow G1 1XJ, Scotland, UK; 3 Department of Mechanical, Mechatronics and Manufacturing Engineering, University of Engineering & Technology, Lahore 548900, Pakistan; amjad9002@uet.edu.pk; 4 Department of Production Engineering, National Institute of Technology, Tiruchirappalli 620015, Tamil Nadu, India; saravana312@gmail.com

**Keywords:** sustainable manufacturing, minimum quantity lubrication, cryogenic machining, solid lubricants, vegetable oils, steels

## Abstract

Machining operations are very common for the production of auto parts, i.e., connecting rods, crankshafts, etc. In machining, the use of cutting oil is very necessary, but it leads to higher machining costs and environmental problems. About 17% of the cost of any product is associated with cutting fluid, and about 80% of skin diseases are due to mist and fumes generated by cutting oils. Environmental legislation and operators’ safety demand the minimal use of cutting fluid and proper disposal of used cutting oil. The disposal cost is huge, about two times higher than the machining cost. To improve occupational health and safety and the reduction of product costs, companies are moving towards sustainable manufacturing. Therefore, this review article emphasizes the sustainable machining aspects of steel by employing techniques that require the minimal use of cutting oils, i.e., minimum quantity lubrication, and other efficient techniques like cryogenic cooling, dry cutting, solid lubricants, air/vapor/gas cooling, and cryogenic treatment. Cryogenic treatment on tools and the use of vegetable oils or biodegradable oils instead of mineral oils are used as primary techniques to enhance the overall part quality, which leads to longer tool life with no negative impacts on the environment. To further help the manufacturing community in progressing towards industry 4.0 and obtaining net-zero emissions, in this paper, we present a comprehensive review of the recent, state of the art sustainable techniques used for machining steel materials/components by which the industry can massively improve their product quality and production.

## 1. Introduction

Manufacturing products while conserving natural resources and causing no negative environmental impacts is called sustainable manufacturing. Manufacturing industries create products for fulfilling human needs; however, this includes the consumption of huge amounts of raw resources and the generation of wastes which are increasing day by day and can be very detrimental for our environment.

The following three stages of product waste are primary factors for waste generation and the degradation of the environment:

- In the manufacturing processes;
- During usage of the product;
- At the end of the life of the product.

The production of metals triggers the consumption of natural resources and has created a harmful effect on humankind. To avoid using resources needed by future generations, it is necessary to use fewer natural resources and reduce the negative environmental impact caused by manufacturing systems. That is why industries are now moving towards sustainable manufacturing. Early ideas about sustainable manufacturing first appeared in the 1970s and 1980s [1,2,3,4,5].

### 1.1. Manufacturing Industries and Sustainable Manufacturing

It is well noted that machining is widely used to produce automotive parts within the manufacturing industry sector. In all machining operations, cutting fluids play a vital role in reducing the machining cost by increasing tool life. It was observed that 7–17% of the cost incurred in the machining of a part is associated with using cutting fluids. Further, the tooling cost is about 2–4%, so it is necessary to improve the whole process. In addition, the use of cutting fluids causes health diseases like skin problems, allergies, eyes problems, and cancer in workers. Here, skin problem is about 80% [6]. Lawal et al. [7] also witnessed that major skin problems, about 80% in quantity, are due to cutting fluids. They also proposed that vegetable oil-based and metal working liquids have been proven to be environmentally sustainable in the dielectric regime.

Strict environmental regulations demand that cutting oil used during machining processes should be recycled or disposed of in such a way that it will not spoil the environment and will be harmless for all interested parties. These fluids are extremely costly to dispose of or store. The cost is about double the machining cost depending on the cutting fluid which is being used. Mineral oils used as cutting fluids are difficult to dispose of into the environment without any prior treatment [8,9,10].

### 1.2. Need for Sustainable Manufacturing

Jordi Oliver Solà, Chief Executive Officer (CEO) of a circular economy consulting group, has demonstrated the importance of sustainability, not only from an ethical or environmental point of view, but also that it is needed for markets to be competitive and important for the survival of any sector [11]. Therefore, to save natural resources, sustainable manufacturing is very important [12,13].

**Figure 1:**
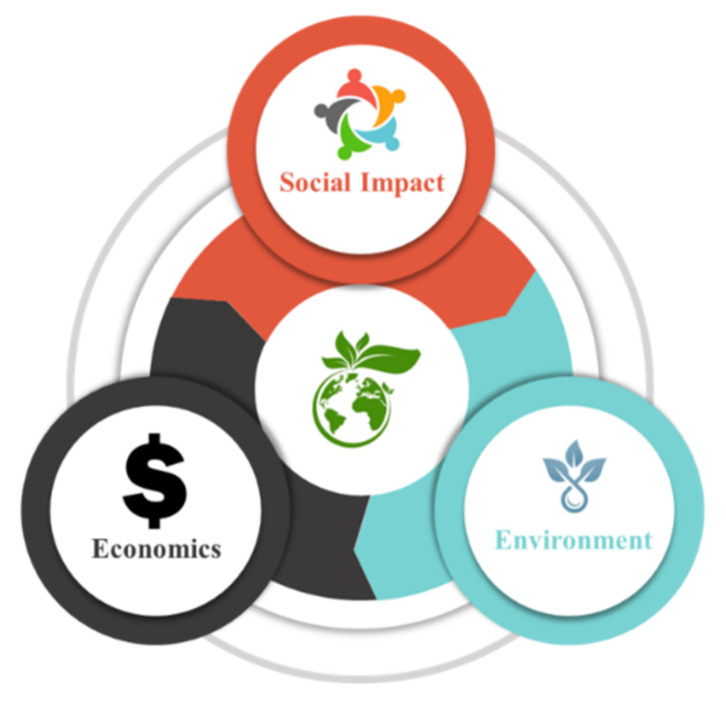
Three Pillars of Sustainability, reprinted with permission from ref. [16]. Copyright 2017 BSP books Pvt Ltd.

The need for sustainable manufacturing techniques is also depicted by the three pillars of sustainable manufacturing, as shown below in Figure 1. One of these is the need for improvement from an economic, social, and environmental point of view. It brings balance between social, economic, and environmental aspects [14,15]. This technique mainly deals with the minimal usage of cutting fluids. It does not mean just stoping the supply of cutting oils to make the environment better. Cutting oils serve many purposes like lubrication and temperature reduction in the cutting zones.

Concerns about environmental impacts, climate change, occupational health and safety, and machining costs have forced companies to move towards sustainable techniques. As per the investigation of Jayal et al. [17], the selection of sustainability aspects occurs mainly because of factors like the increase in diseases in shop floor workers, inflexibility in government plans, and when targeted to minimize the cost of production. So, sustainable machining is highly recommended where traditional cutting methods became null. Currently, advanced technologies like cryogenic cooling, nano cutting fluids, dry cutting, and minimum quantity lubrication (MQL), etc. are being used [18].

Several studies have been published that emphasize the importance of sustainability. For example, Zein et al. [19] presented certain resources which are associated with the manufacturing technologies, including production tools and methods that directly correlate with economic impact. They outlined that the sustainability of a firm can be affected by manufacturing approaches. Jayal et al. [17] established a case study on machining techniques by improving the model at the process, product, and system-level for sustainable manufacturing. Sarkis [20] found a relationship between environmental concerns and manufacturing activities. The study concluded that sustainable machining not only deals with environmental initiatives but also included techniques that empower benefits for humanity. Lu et al. [21] developed a metric to ensure the sustainability of the manufacturing process. They also established the interrelationship between the elements of metrics and studied the potential impact. The metrics broadly covered the given elements, i.e., social, environmental, and economic. Jawahir and Dillon [22] and Hegab et al. [23] highlighted six of the most important factors that alter the paradigm of sustainability in manufacturing processes. The factors included cost, energy, the safety of workers, personal health, environmental impact, and waste. The researchers said that out of the six aforementioned elements, waste, cost, and energy can be more easily computed than the rest of the elements. Waas et al. [24] made a framework for the sustainability of manufacturing sectors by taking the hierarchy of social needs and combinations. Then they used the Delphi technique to propose the metrics for each category. Some researchers suggested rules to achieve sustainability in the manufacturing firms, as demonstrated by Lovin et al. [25]. The rules are (1) minimal usage of energy and material, (2) usage of cleaner production, recycling and conversion techniques to reusable substance, (3) adoption of a solution based system (i.e., supply chain structure) rather than a proactive business model, and (4) reinvestment in natural substitutions that are available for distinct materials, such as investing in renewable resources instead of non-renewable substances. From the machining perspective, Diaz Elsayed et al. [26] discussed a detailed study about the combination of green and lean in the automotive organization. The purpose of their research was to determine the effect of green-lean in the manufacturing sector. They concluded that grouping of green-lean proved an effective way of improving manufacturing firms in terms of waste reduction, less resource utilization, and energy consumption. Thus, they stated that the use of green-lean is a sustainable manufacturing approach for different enterprises. Abdul Rashid et al. [27] also investigated environmental performance by employing sustainable manufacturing techniques. They proposed that the main environmental initiatives are entirely based on manufacturing practices. In the same vein, Rusinko [28] established the relationship between manufacturing activities and their results. The results revealed that manufacturing cost is decreased by preventing waste and unnecessary substances. According to Gimenez et al. [29], organizations should improve their environmental, social, and economic behavior to get a sustainable approved system. The aforementioned literature divulged the importance of sustainability in manufacturing or business firms. Therefore, the current study was conducted to scrutinize a systematic review of the sustainable machining of steel, as it is being used in scattered application areas including aerospace, automotive, nuclear power plants, and medical equipment, etc.

There are numerous benefits, i.e., financial, environmental, and safety, which are related to the three pillars as discussed before. The need to turn towards sustainable manufacturing is due to many reasons like occupational health-related problems, environmental regulations, and unsafe or polluted environments for workers, but the largest is the waste cost in using too much cutting fluid in metal cutting industries. In such industries, the costs associated with purchasing, maintaining, the makeup of cutting fluid, cutting oil, and system cleaning are more prominent.

The consideration of the following points allows companies to improve these three pillars:

- Efficient resource utilization (Energy, Material, Water, Labor, etc.);
- Improvement in the application of metalworking fluids;
- Adopting other sustainable manufacturing techniques;
- Lean Implementation;
- Improvement in the working environment by applying best machining practices;
- Most important, training to all employees related to sustainable machining.

**Figure 2:**
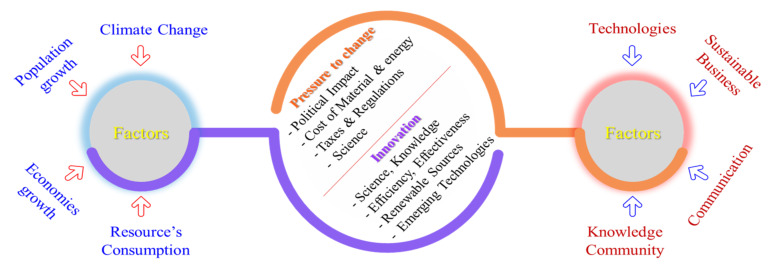
Pressure to change the paradigms of the manufacturing industry, reprinted with permission from ref. [30]. Copyright 2010 Elsevier Ltd.

**Figure 3:**
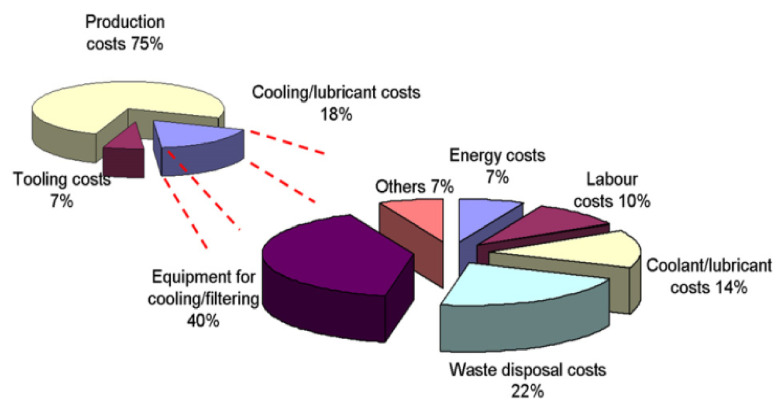
Cooling and lubricating costs incorporated in the automotive sector, reprinted with permission from ref. [31]. Copyright 2010 Elsevier Ltd.

Figure 2 shows the basic objectives by which pressure was built on manufactures to change their way of working. It is clearly depicted that there is a need to change the whole scenario of conventional working in manufacturing industries to improve socially, economically, and environmentally. Technology revolution should be introduced in manufacturing industries to lower the cost per piece of product. Whereas Figure 3 shows the breakdown of the product cost, including cooling and lubricating costs, which are about 18%.

**Figure 4:**
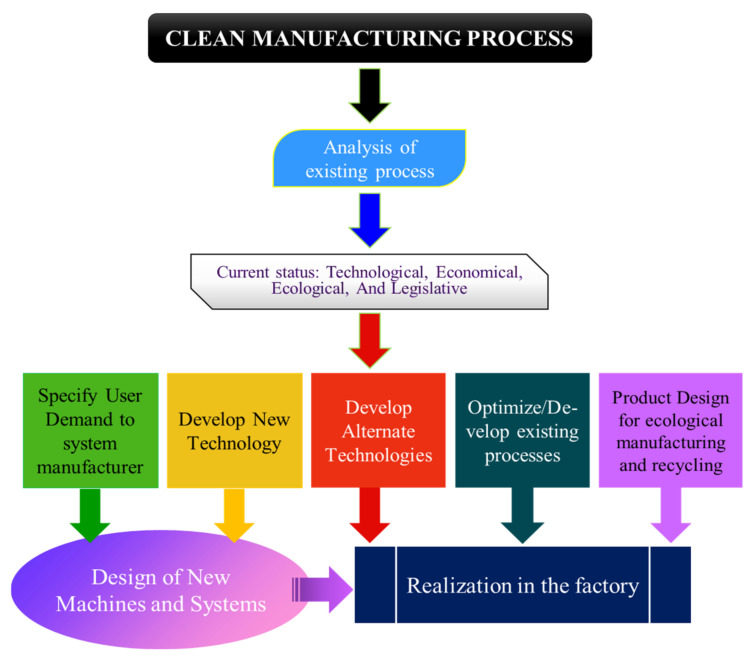
Implementation of Clean Manufacturing process, reprinted with permission from ref. [31]. Copyright 2010 Elsevier Ltd.

Manufacturing companies can improve their costs and tackle environmental issues by implementing sustainable principles. Implementation can be done by analyzing the current situation of the process or system in any industry. There is a need to adopt alternate technologies for redesigning systems for the effective realization of these principles in factories [30]. The key methods that provide a direct path to create a cleaner manufacturing sector are depicted in Figure 4.

**Figure 5:**
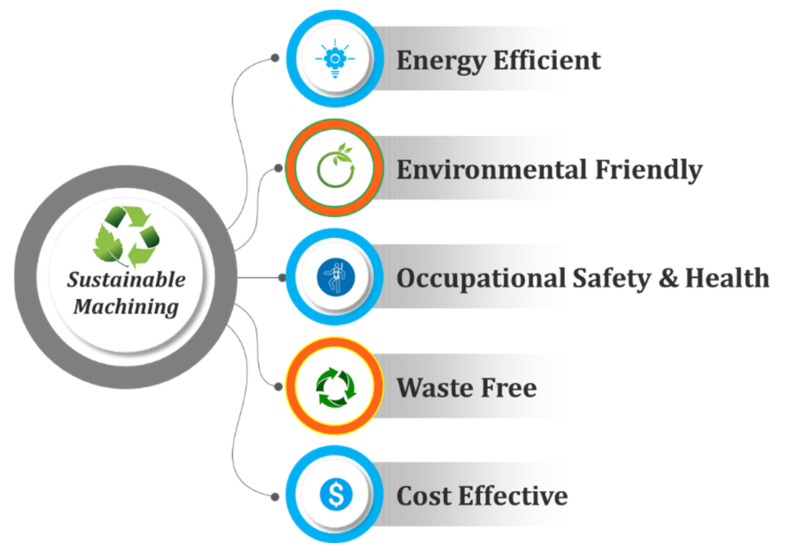
Characteristics of Sustainable machining, reprinted with permission from ref. [17]. Copyright 2010 CIRP, Published by Elsevier Ltd.

**Figure 6:**
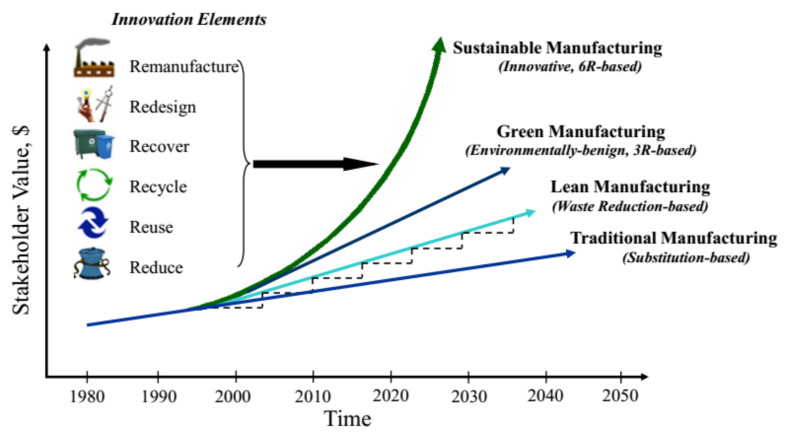
Evolution with the time of sustainable manufacturing, reprinted with permission from ref. [32]. Copyright 2010 CIRP, Published by Elsevier Ltd.

Further, some key characteristics of sustainable machining are presented in Figure 5, which clearly shows that this technique justified three pillars of sustainable manufacturing. Figure 6 shows the evolution over time of sustainable manufacturing, which depicts the critical importance of embedding sustainable manufacturing by 2025. It is assumed that the industries will work on 6-reduction (6R) elements rather than 3-reduction (3R) entities which are used in the actual green manufacturing model [32].

As per the given literature, it has been found that certain areas need to be reviewed. For example, material wastage and the amount of disposed-off material during the process may cause the cost of machining to be high. Moreover, suspended particles enter the environment and damage the quality of the air. The disturbing air quality index influences human life, and thus sustainability is compromised. Therefore, this review has been developed to understand the conditions/parameters of different sustainable machining techniques which alter the cost, pollute the environment, and decrease the overall productivity. For this context, a PRISMA approach has been adopted to study the variants of sustainable manufacturing processes as far as steel material is considered. The current review is restricted to sustainable techniques used for the machining of steel in order to ensure cost-effective, environmentally stable, and eco-friendly processes.

Even though this review provides a comprehensive discussion on sustainable manufacturing techniques, other elements may be added, like frostbite hazards in cryogenic machining and initial setup cost, which is difficult to afford by any local industry. Therefore, a comprehensive investigation is required to mitigate the aforesaid issues of cryogenic machining by ensuring a controlled temperature environment. The mathematical modeling of the sustainable cutting mechanisms with respect to the cutting of steel is still an area that needs special focus.

This paper is presented in the following order: (i) A brief introduction with mechanical properties of some steel grades is proposed in Section 2. (ii) The comprehensive methodology is given in Section 3. (iii) Different sustainable techniques employed in a couple of manufacturing sectors are demonstrated in Section 4. It constitutes different subsections; each outlines the discussion, significance, advantages, disadvantages, and limitations of each sustainable technique separately. (iv) The detailed discussion about the present work, along with comparisons between each sustainable technique, has been granted in Section 5. (v) Section 6 illustrates the multiple challenges faced with the implementation of sustainable manufacturing. (vi) Fundamental issues associated with additive manufactured steel has been given in Section 7. (vii) The findings are summarized in Section 8. (viii) Finally, future implications have been revealed in Section 9.

## 2. Steels’ Classification, Properties, Machining Difficulties, and Sustainability Requirements in Steels’ Machining

**Figure 7:**
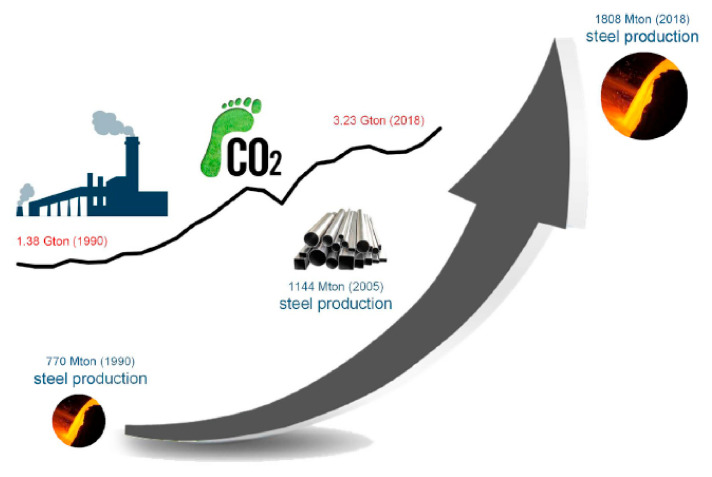
Worldwide steel production in 2018, along with the CO_2_ emission, reprinted with permission from ref. [34]. Copyright 2019 Elsevier Ltd.

**Figure 8:**
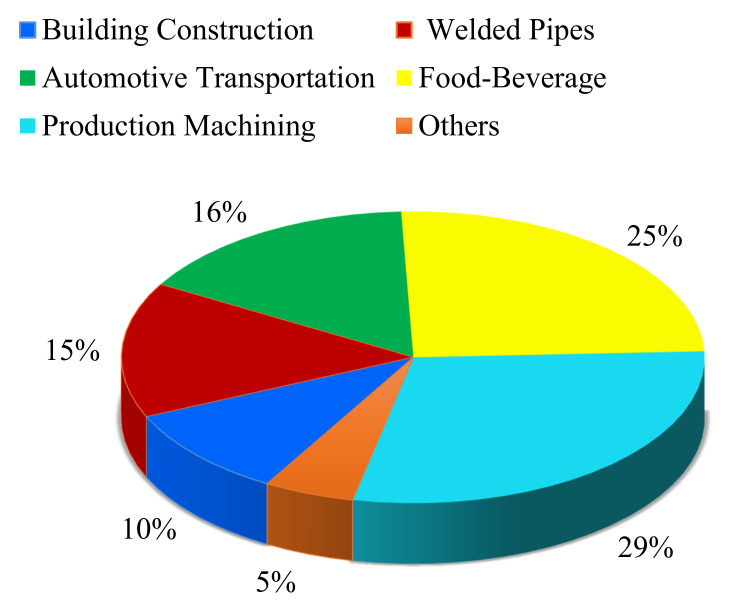
Division of steel usage in different applications.

An alloy of iron (Fe) with minimal carbon content is referred to as steel. Carbon (C), generally up to 1.5%, is present in steel [33]. As per the literature, about 1808 million tons of steel were produced in 2018 worldwide. This is depicted in Figure 7, along with the emissions of CO_2_ [34]. If the machinability perspective of steel is under consideration, then up to 29% of steel is employed in machining, as given by Diva Metal Ltd. Company [35]. Figure 8 represents the division of steel in different applications. Steel exists in the form of different variants like structural steel, heat resistant steel, and tool steel, etc. Another important class of steel is named Alloy Steel, a standard form that constitutes various elements (i.e., nickel, magnesium, copper, titanium, vanadium, silicon, boron, and manganese, etc.) in different proportions that range from 1.0% to 50% by weight. Alloy steel can be categorized into low alloy steel (LAS) and high alloy steel (HAS). Usually, the phrase “alloy steel” is related to LAS. Nickel (Ni) is a prime element in LAS that has the ability to increase the strength and ductility of different engineering applications, including jet engines, spacecraft, and nuclear reactors. Interestingly, Ni also amplifies the characteristics of ferrite steel, such as stability at low-temperature toughness, which allows them to be used in cryogenic applications [36]. For instance, steel with 9% Ni can be employed for liquefied natural gas (LNG) handling and storage purposes. Moreover, it assists in nitriding, carburizing, and tool steel due to tremendous properties like good strength, high hardness, superior toughness, the ability to withstand elevated temperatures, excellent wear, and corrosion resistance. The other combination, such as an alloy of Fe with C, is known as the simplest alloy. The ferromagnetic feature of Fe permits the use of the simplest steel in magnetic applications like electric motors, transformers, etc. [37]. The details about some key classes of steel are presented in the forthcoming sections.

### 2.1. Structural Steel (SS)

SS is a commonly used building material in the construction industry. The performance of SS is now predictable and depends on standards recognized by the American Institute of Steel Construction (AISC), which elaborate shapes, sizes, elemental composition, as well as mechanical attributes. SS is 100% recyclable and has proven to be one of the most reprocessed materials in the world [38]. The fundamental classification of structural steel is:

i. Carbon-manganese steel;
ii. High strength low alloy (HSLA) steel;
iii. High strength quenched and tempered alloy steels.

From the above-mentioned classes of structural steel, HSLA is important because it provides good mechanical properties, high resistance to rust, and high weldability with a carbon percentage between 0.05–0.25%. The other benefits of HSLA steel are: (i) light in weight, (ii) good strength to wear ratio, (iii) and control over internal and external stresses. However, HSLA has limitations in terms of acquiring more power (>25–30%) as compared to carbon steel. Additionally, such steel has sensitivity in directional properties [39]. The chemical composition of HSLA-80 steel is presented in Table 1, whereas the mechanical properties are listed in Table 2.

### 2.2. Heat Resistant Steel

Heat resistant steels (HRS) are a unique class of steel alloys that can easily be operated at temperatures as high as 750 °C. To attain their specific properties, all HRS are composed of numerous elements, two of which are considered basic elements, i.e., Chromium (Cr) and Nickel (Ni). Cr is preferred for corrosion resistance, while Ni is useful to obtain high strength and ductility. The other elements (aluminum, cobalt, manganese, niobium, copper, zirconium, and phosphorous, etc.) are added to achieve high-temperature properties along with good weldability. Based on chemical stability, high strength, and superb corrosion resistance, HRS are divided into three types; (1) Low alloy steels, (2) Martensitic steels, (3) Austenitic steels [40].

Low allow steels are extensively used in pressure-based applications like steam boilers and thermal power plants due to unique characteristics such as mechanical strength, great toughness, and sufficient rust resistance ability. Such steel alloys are mostly preferred in thick components such as headers, pipes, and control valves. The different grades of low alloy steel have applications in distinct areas. Grade 11 (1CrMoV) and 22 (2.25Cr1Mo) are used in power developing industries. The mechanical properties of these low alloy steels are tabulated in Table 3. In the same way, Fe-0.1C-xMn and Fe-0.1C-xNi, where x = 1.5, 3% by mass, are special kinds of low alloy steels used for cryogenic treatments to decrease the corrosion property as well as improve the microstructure. The chemical composition of all the said low alloy steels are mentioned in Table 4.

Martensitic heat resistant steels contain medium and high chromium contents of about 5–9% Cr and 12%, respectively. They have been fabricated for power plant materials where the temperature is significantly higher, such as 650 °C. The high percentage of Cr in such steel alloys enhances the creep strength and corrosion resistance of the materials because of a low coefficient of thermal expansion and high thermal conductivities in contrast to austenitic steels. Moreover, the emission of hazardous fumes and gases and the efficiency of the power plant are also raised due to the application of Cr. 9Cr and 12Cr, a special series of martensitic alloy steels [40]. The elemental composition of some martensitic alloys is given in Table 5, and mechanical properties are elaborated in Table 6.

Austenitic steel is also called austenitic stainless steel, with a Cr percentage of about 13% by weight at room temperature. The high cost associated with these alloys is due to the high percentage of supplementary elements compared to other steel alloys. The applications of austenitic alloys are limited to those conditions where chances of corrosion are substantial, such as in boiler tubes. They have similar properties to martensitic steels, except high thermal loading can lead to wear and tear over the surface. The FeCrNi is the most commonly used austenitic steel. However, AISI (American Iron and Steel Institute) 302, 304, 321, 347, 316, 309, ASS304L, ASS316L, and other alloys are also employed in different application sectors. The elemental composition of few austenitic alloys is provided in Table 7.

### 2.3. Tool Steel

Tool steels are alloy steels that are appropriate for the manufacture of tools due to their excellent properties like high hardness, low deformation, minimal abrasion, and no wear and tear, even at elevated temperatures. Apart from the mentioned properties, these steels have a high magnitude of tensile and compressive yield strength which tends to minimize the plastic deformations at the stress concentration points in the tooling [47]. There are different variants of tool steels, including cold working, hot working, high speed, vibration resistance, water hardening, and some unusual purposes. The selection of this group is based on cost, temperature, surface hardness, ductility, and toughness values. In severe circumstances, carbide tool steels are utilized. They have applications in cutting, drawing dies, pressing, cold extrusion dies, broaches, thread rolling, forming rolls, and coining of materials. Another important application of tool steel is in the injection molding process, where durability plays an integral role. The common scale of tool steel grade is AISI-SAE. The chemical composition of some tool steel is given in Table 8.

Different machining methods have been practiced in past investigations on the steel material, including milling, drilling, broaching, grinding, planing, and turning, etc. However, some methods induced complications while machining steel materials. For instance, Nagy et al. [49] said that machining (turning operation) of super duplex stainless steel is highly challenging when cutting tool inserts made up of PVD coating are used. The difficulties may be due to continuous and long chips formation, which is often problematic in the context of chip handling. Furthermore, long chips rolled on the part, and accordingly, stimulates surface imperfections. Eventually, surface quality is compromised. A similar problem has engaged with the austenitic steel. Sunil Magadum et al. [50] claimed that high strength, greater toughness, large fatigue, and corrosion resistivity are the prime reasons behind the poor machinability of steel. All the stated factors cause build-up-edge, irregular electrode wear, early tool failure during cryogenic machining of SS304 steel. Ingle et al. [51] proposed that there are certain grades of steel that have a machinability rating of 40%. Those grades belong to the austenitic steel such as 302B, 309, 309S, 330, 384, and 314. The authors demonstrated that a rating of less than 100% refers to the difficulty of machining alloys. As long as a rating is going down, then the difficulty level raises accordingly. The issues attributed to the aforesaid grades of austenitic steel are characterized by high ductility, toughness, prolong work-hardening, and less thermal conductivity. The machinability issues of martensitic steel grades (414, 422, 431, 440A, 440B, and 440C) has also been discussed for the austenitic steel grades.

Steel materials are the most used materials in designing and manufacturing automotive components and in several other industrial sectors. The growth of the manufacturing industry, together with the need for cleaner production, makes the integration of sustainable techniques necessary. To help the manufacturing sector and research community find the best option to meet these goals, we present in this work a detailed review of major sustainable techniques used in the manufacturing of steel materials. Further, the details presented in this review can act as a guide in selecting the best solution to be integrated towards achieving net-zero emissions in their manufacturing process.

Numerous studies have been presented on the various grades of steel. Laleh et al. [52] demonstrated the unexpected behavior of LPBF 316L in the context of erosion and corrosion. They proposed that lower erosion and corrosion resistance of the selected austenitic stainless steel is due to its minimum repassivation through traditional techniques. Thompson [53] contrasted HSLA-80 steel with two alternatives of HSLA, i.e., HSLA-80/100 & HSLA-100, considering yield strength, fracture, and results of Charpy impact test. They found that outcomes of yield strength are enough to study the microstructure, as long as strength and toughness are concerned. Durmusoglu et al. [54] joined the HSLA-80 steel by employing gas metal arc welding based on the high strength of weld metal followed by the heat-affected zone (HAZ) and target metal. Furthermore, the author detected that martensite needle-like sand is looked up in the HAZ, whereas the weld metal has residual austenite. Rajbongshi et al. [55] analyzed the effect of the surface topology of AISI D2 steel at the flank side using texturing and non-texturing coated carbide tools. Two responses (flank wear and surface integrity) were evaluated against three factors, i.e., speed, feed, and depth of cut. The results predicted that texturing tools yield minimal flank wear and less surface roughness (SR). Rath et al. [56] investigated the effect of dry machining on the newly developed grade AISI D3 steel using a mixed ceramic insert (Al_2_O_3_ + TiCN). Three control parameters (cutting speed, feed rate, and depth of cut) were used to evaluate the influence on cutting forces, SR, electrode wear, and chip thickness. They revealed that feed rate is the most dominant factor, which alters the magnitude of all the defined responses magnificently. Kajendirakumar et al. [57] also conducted a study on AISI D3 steel. They optimized the process parameters via the electric discharge machining (EDM) technique by utilizing grey relational analysis. Material removal rate (MRR) and SR were taken as output responses. They said that optimum parameters were achieved at low pulse on time, high pulse off time, and a large value of current. Guo et al. [58] studied the microstructure and characteristics of heat resistant steel (2.25Cr1Mo0.25V) using the Wire-Arc AM (WAAM) process. They claimed that subtract after processing through the WAAM technique exhibit high quality, excellent metallurgical features, and defect-free surface. Baddoo [59] has proposed a review article about the challenges, applications, and opportunities of stainless steel in the construction sector. The author stated that stainless steel had been proven to be a good alternative in construction sectors because of its good mechanical strength and high ductility. However, these are also fundamental requirements of any architectural applications. Ramana et al. [60] depicted the influence of powder (Nickel) contained EDM on MRR, tool wear rate (TWR) using die steel material against copper electrode. They estimated that nickel in dielectric fluid substantially improves both the said output when pulse-on/off time and current are considered as input variables.

## 3. Methodology

**Figure 9:**
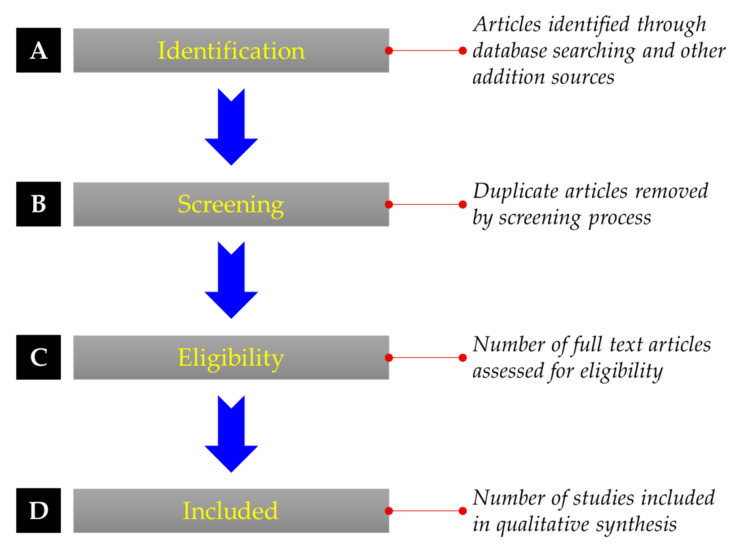
A PRISMA Methodology, reprinted with permission from ref. [61]. Copyright 2009 BMJ Publishing Group Ltd.

This module describes a detailed methodology for the sustainable machining of steel that has undergone a comprehensive review procedure. A PRISMA (Preferred Reporting Items for Systematic Reviews and Meta-Analysis) approach was adopted, as displayed in Figure 9, to study the multiple intents of sustainable techniques [61]. The different aspects of sustainable machining techniques for the steel material comprise processing, benefits, drawbacks, and limitations. Afterward, the three pillars of sustainability, such as social- environment -economic, are critically reviewed and highlighted during steel machining to find out the research gaps and future implications. From this perspective, different literature has been studied from various Journals, including Science Direct, Tandfonline, MDPI, Springer, Hindawi, Wiley, Web of Science, etc. The iterative forward and backward strategy was practiced in the identification process to collect the explicit information using the Keywords, Sustainable manufacturing, MQL, Cryogenic machining, Solid lubricants, Vegetable oils, and Steels.

For the acquisition of Journal articles, books, reports, and web pages, the string of sustainable machining was utilized in each of the databases’ searches. Then a screening operation was performed to find out the future implications in sustainability machining of steel by appraising the existing issues and tentative solutions in a contextual manner. Each of the content of the research articles has been extensively examined while taking the sustainability viewpoint of steel into account. Based on the following established criteria, a large amount of information taken from published literature was systematically organized for assessing future research possibilities:

- Studies belonging to human health, environmental and economic impact on the machining of steel;
- Investigations related to the mechanical and chemical characterization of the machining under special cutting oils/fluids;
- Articles linked with the MQL machining attributes of steel material plus the cryogenic treatment cutting effect on the suitability of steel;
- Content affiliated to the behavior of dry machining of steel.

The references have been cited within a broad time span from 1982 to 2021. Out of the complete list of references, about 43% of articles have been selected from the last six years (2015–2021). The fundamental information based on the sustainable machining techniques for steel was collected and organized, then sub-categorized as per the importance in the respective studies. A comprehensive revision of the research records was developed to examine the sustainability aspect of steel, keeping an eye on its machining attributes. In the present study, challenges to the sustainable machining of steel were also described, and the discussion of this research is summarized in the Conclusion. Finally, future directions and research limitations have been consolidated using the identified knowledge about the sustainable machining aspect of steel.

## 4. Sustainable Techniques

**Figure 10:**
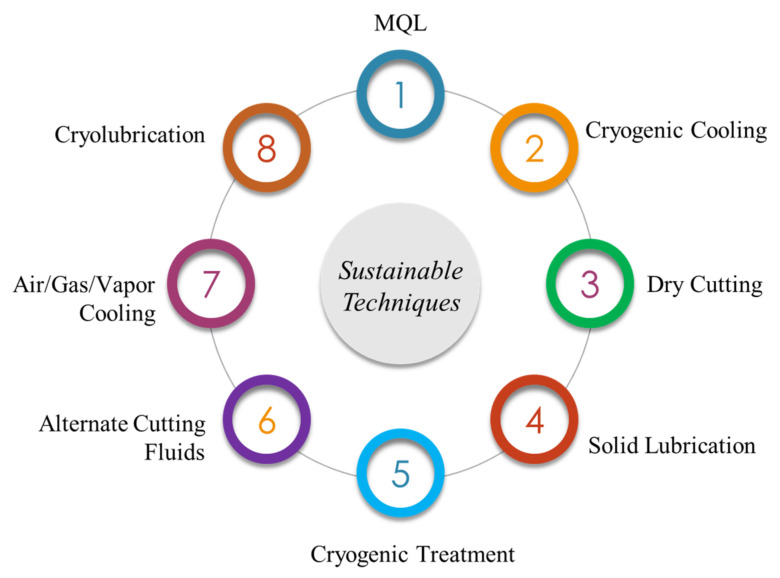
Sustainable Techniques, reprinted with permission from ref. [8]. Copyright 2015 Elsevier Ltd.

**Figure 11:**
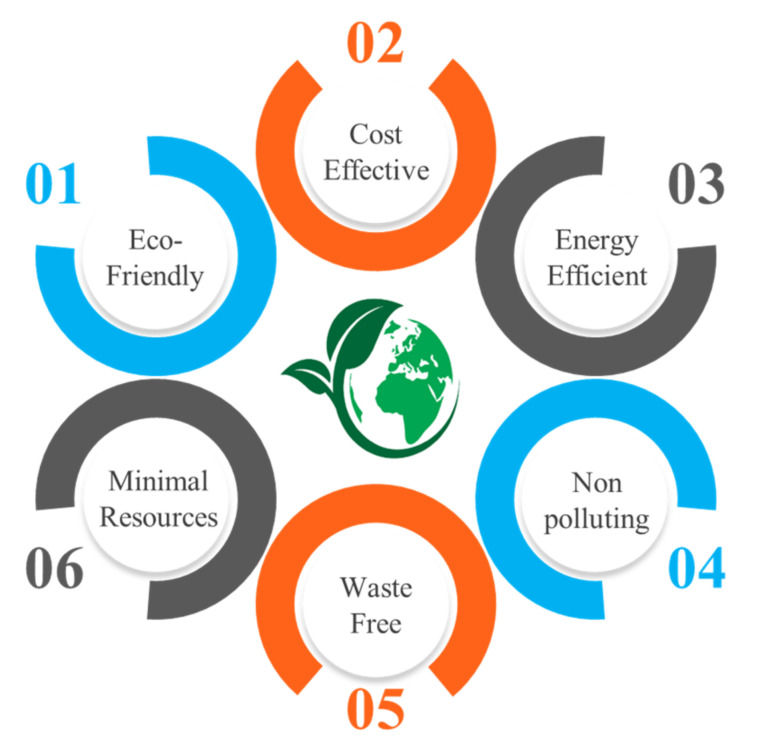
Benefits associated with sustainable machining.

Different sustainable techniques, i.e., cryogenic cooling, MQL, solid lubricants, and other techniques which are being used in the auto industry that fulfill the overall objectives of this review, are depicted in Figure 10 [8]. The techniques mentioned in Figure 10 have certain benefits, as portrayed in Figure 11.

### 4.1. Cryogenic Cooling

**Figure 12:**
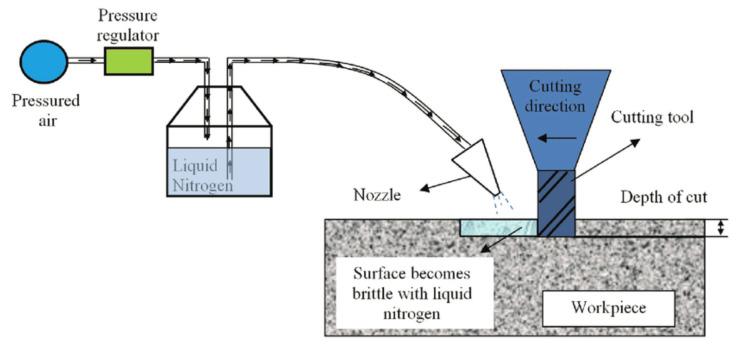
Diagram of cryogenic cooling technique setup, reprinted with permission from ref. [64]. Copyright 2010 Elsevier Ltd.

In cryogenic cooling, low temperature (below −150 °C) materials and medium are used for cooling purposes. Liquid nitrogen, whose boiling point is (−195.82 °C) and frozen carbon dioxide, whose sublimation point is (−78.5 °C), are two common media used in this process. Nitrogen is employed to cool down the temperature in the cutting zone because of exothermic conditions. The large amount of heat that is generated during machining causes tool failure and tends to alter the mechanical properties of the specimen. Therefore, to minimize the detrimental effects due to heat and elevated temperature, nitrogen is used, which decreases wear and tear as well as improves the build-up edge [62]. Cryogenic is an eco-friendly technique that shows better results at higher cutting speeds. It is best to control machining temperature along with enhanced tool life [63]. The schematic diagram of the cryogenic cooling setup is represented in Figure 12.

It was noted during the comparison of dry cutting, MQL, and cryogenic machining that the cryogenic technique is better in increasing tool life with the reduction of cutting temperature. With this product, life improved due to better surface quality [65].

The use of liquid nitrogen in hard turning caused the improvement in cutting speed, and higher productivity and greater tool life were achieved. All of the surface finishes also improved as it causes a decrease in machined surface temperature. Also, it is good for the environment and has no toxic properties [66].

**Figure 13:**
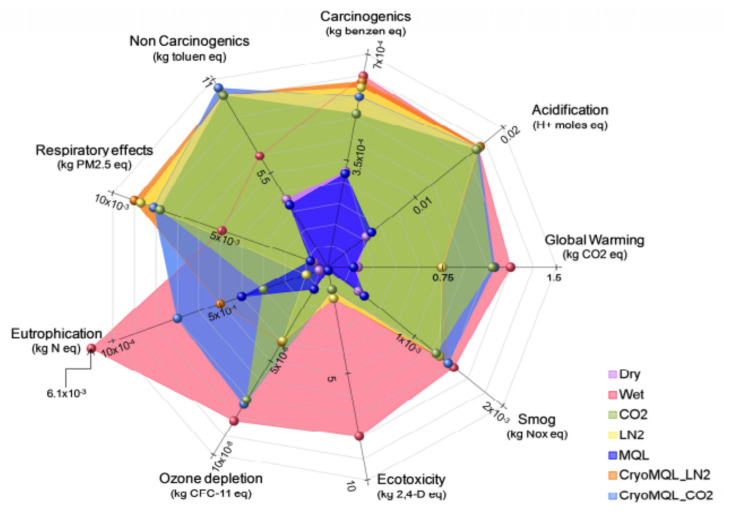
The impact on the environment by different cooling techniques, reprinted with permission from ref. [67]. Copyright 2016 Elsevier Ltd.

Figure 13 shows the environmental impact of different cooling techniques in the machining of AISI 304. Wet cooling has a tremendous impact on the environment, like ozone depletion, etc. Cryo MQL-CO_2_ is best found in all these.

**Figure 14:**
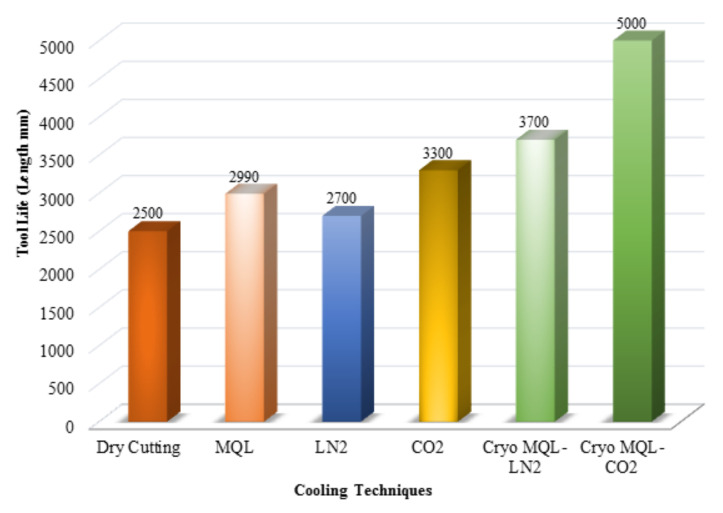
Comparison of Tool life between different cooling techniques, reprinted with permission from ref. [67]. Copyright 2016 Elsevier Ltd.

It was observed that cryogenic machining, which is suitable for environmental impact, may also have other benefits in terms of lesser tool life and low power consumption as compared to dry cutting. Figure 14 shows a graphical representation of tool life in different cooling techniques, which clearly depicts that tool life is longer in CryoMQL-CO_2_ as compared to other techniques [67]. In the milling of hardened AISI D3 steel, the effect of cryogenic cooling (liquid nitrogen) was noted for tool life, surface roughness, and cutting forces. Cutting forces were reduced by 20% to 27%, and surface roughness was decreased up to 16 to 29% due to less cutting temperature at the tool chip interface. Tool life was increased up to 26% to 35% as compared to dry cutting conditions [68].

The cryo-cooling process consists of many input variables: cooling rate, soaking time and temperature, tempering temperature, and its required time [69]. Gill et al. [70] flourished that three of above parameters (cooling rate, soaking time, and soaking temperature) have been extensively increased the tool life upto 98% by compromising the mechanical characteristics of it. Stratton [71] put forward that cooling rate must be low enough to avoid cracking and deforming in the material. Molinari et al. [72] reported about soaking time that must be less than 35 h. It also stated that tool fracture mainly because of insufficient cooling rate, so the optimum value for cooling rate should be near to 30 °C/h. Barron [73] had observed the effect of soaking temperature (189.15 K and 77.15 K) on the wear resistance property of M2 Steel. Besides, many researchers witnessed that increase in hardness, toughness, improving stability and resistance to corrosion is enhanced the tool life [74,75]. Dhar and Kamruzzaman [76] have compared the dry, wet and cryogenic techniques for AISI-4037 Steel. They concluded that cryogenic has been proved as sustainable method followed by dry and wet method in terms of reduction in heat upto 673.15 K. SR is another important criterion to check whether the machining is sustainable or not. Rotella et al. [77] carried out machining under dry, wet and cryogenic condition on Ti-6Al-4V. They noted that cryogenic machining is more prominent than dry and wet machining in term of getting high surface integrity. They also summarized that cryogenic machining has been proved as effective at high feed rates. Kumar and Dhananchezian [64] also demonstrated the similar consideration about SR in cryogenic machining of Ti-6Al-4V. The 35% improvement in SR magnitude has been observed in comparison to dry and wet processing.

**Figure 15:**
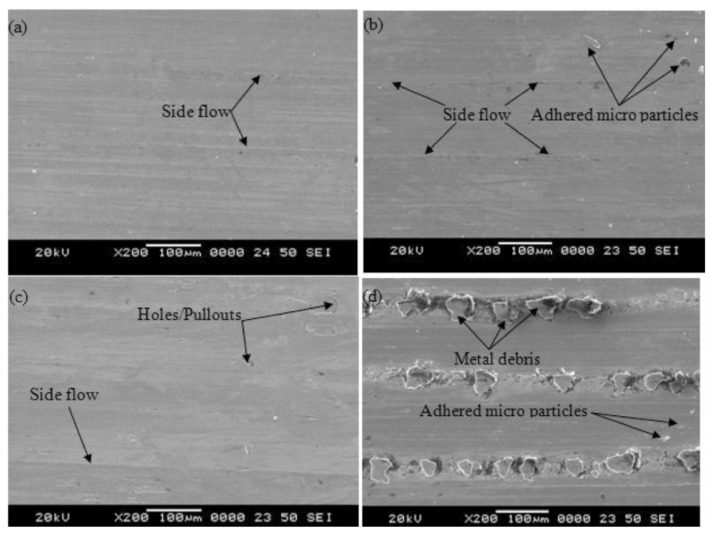
Images of surface morphology under different cooling environments. (**a**) cryogenic, (**b**) wet, (**c**) MQL, (**d**) dry, reprinted with permission from ref. [78]. Copyright 2018 CIRP.

In the turning of 17-4 PH SS, different cooling techniques were used like cryogenic, MQL, and wet and dry turning. Different depth of cut (DOC) was used to check the optimum conditions for each technique. It was noted that the cryogenic technique was best in terms of cutting zone temperature decrement, improved surface integrity, and less tool wear. Chip thickness was also less, and also this technique was environmentally friendly. Figure 15 shows the surface morphology obtained after applying different cooling techniques. The surface was smoother in cryogenic as compared to dry machining [78].

**Figure 16:**
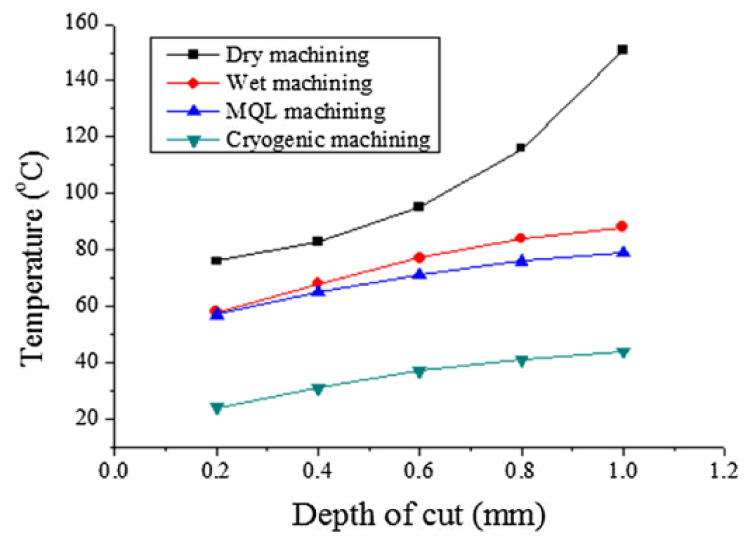
Effect of DOC on cutting temperature under different cooling techniques, reprinted with permission from ref. [78]. Copyright 2018 CIRP.

Figure 16 shows the cutting temperature according to the depth of cut increment, which is lower in cryogenic machining than dry, wet, and MQL machining. In machining AISI 52100 Bearing steel, the effect of cryogenic coolant compared to dry cutting on surface integrity was observed. It was noted that residual stresses and white layer formation were less. This layer is non-recommended because it causes fatigue of the product and affects its life. It became evident that it enhances the surface integrity of hard components in many aspects [79]. In hard turning of 17-4 PH stainless steel, the effect of cryogenic machining was found to be positive. It reduced the cutting temperature by using liquid nitrogen as a cooling medium, and it is eco-friendly. This method can be effectively used in any type of hard material [80].

Nitrogen is most commonly used as it is a safe, noncombustible, noncorrosive gas. The air we breathe has 78% nitrogen gas in it. Liquid nitrogen has the property of easy evaporation, so when it is used in cryogenic machining, it evaporates quickly, and no wastes remain on surfaces, tools, and machines, etc. It contributes to cost savings by avoiding disposal costs [81]. Currently, cryogenic turning is being used to achieve deformation-induced surface hardening. For such purposes, the powerful coolant CO_2_-snow is used due to its good wetting behavior [82].

**Figure 17:**
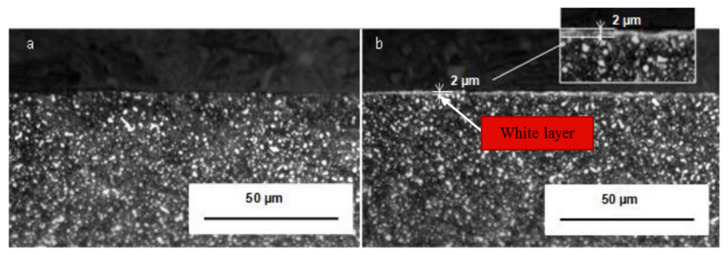
After turning with VNGA160408 (Negative). (**a**) Dry machining; (**b**) CO_2_ machining, reprinted with permission from ref. [83]. Copyright 2015 Elsevier Ltd.

**Figure 18:**
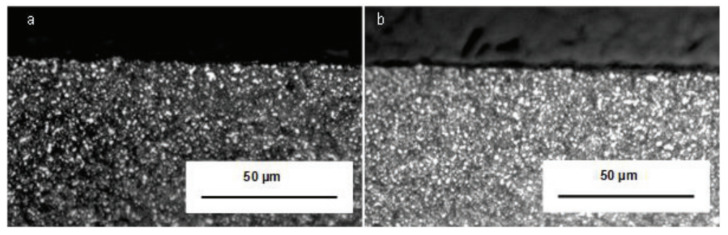
Microstructure after turning with VCGW160408 (positive). (**a**) Dry machining; (**b**) CO_2_ machining, reprinted with permission from ref. [83]. Copyright 2015 Elsevier Ltd.

In hard turning of ASP23 steel, CO_2_ cryogenic media was used with two types of inserts: one negative and one positive. Tool life was increased in the negative insert up to 19.96%, but in the positive insert, the value of improvement rose to 69.5%. The white layer was also checked. In the negative insert during CO_2_ cryogenic machining, it produced a minimal thickness of 2 micrometers. In the positive insert, this layer was not produced. In Figure 17, the microstructure of the material in which machining is done with negative insert using both techniques: dry turning and CO_2_ machining [83], is shown. In Figure 18, the microstructure is presented in which machining is done with positive insert using dry turning and CO_2_ machining. The white layer is not produced, which indicates good structure.

**Figure 19:**
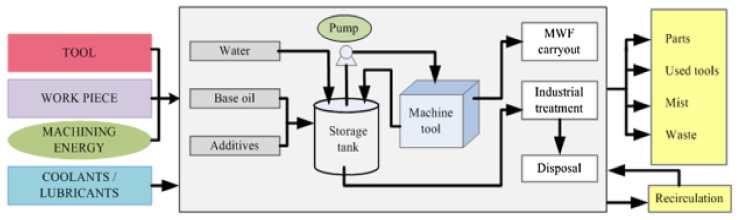
Machining, reprinted from ref. [86]. Copyright 2014 Elsevier Ltd.

In hard turning of AISI 420 steel, the effect of cryogenic cooling was noted compared to nano fluids. It was noted that tool life at a cutting speed of 75 m/min was increased by approximately 29%. This effect was increased as the speed was increased. Also, the temperature is reduced as compared to nano fluids. Chip morphology was better than nano fluids. It was noted that tool wear was also less [84]. In the machining of AISI 4340, it was found that cutting powers are reduced in cryogenic (LN2) cooling as compared to other water-based cutting fluids. Material removal rate (MRR) was increased with a decrement in surface roughness, which was 0.97 micrometers in cryogenic cooling [85]. The comparison is shown between conventional machining and cryogenic machining. In Figure 19, a conventional machining setup is shown in which cooling and lubricants are required, and waste is generated.

**Figure 20:**
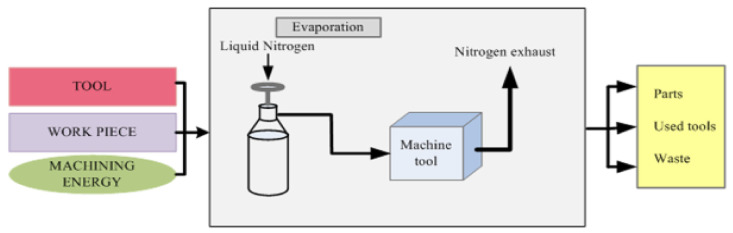
Cryogenic Machining, reprinted from ref. [86]. Copyright 2014 Elsevier Ltd.

In Figure 20, a cryogenic setup is shown. Unlike conventional machining, there is no need for lubricants, and no waste is generated, which is better for the environment and saves on the cost of the product. To observe the cryogenic effect in hard turning of AISI 4340, a setup was done on the shop floor of the CNC turning center. By this process, surface roughness was achieved up to 0.4 micrometers. Tool life of order was achieved 34 min. Cutting forces were reduced by 18%, and power consumption was decreased by about 320 W.

**Figure 21:**
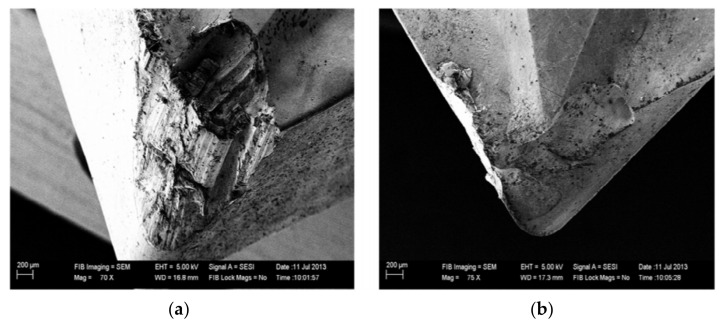
Images of (**a**) wet cooling insert (**b**) cryogenic machining insert, reprinted with permission from ref. [87]. Copyright 2017 Elsevier Ltd.

In Figure 21, an SEM image was captured to check the flank and rake area of the cutting insert after machining. The insert was chipped off when flood cooling was used while in cryogenic machining abrasion type phenomenon observed at flank face [87].

It was noted that power consumption in terms of electricity creates about 99% environmental impacts, which need to be minimized. It was done by choosing the optimal cutting conditions in terms of CO_2_ emission, which leads to better environmental impacts [88].

**Figure 22:**
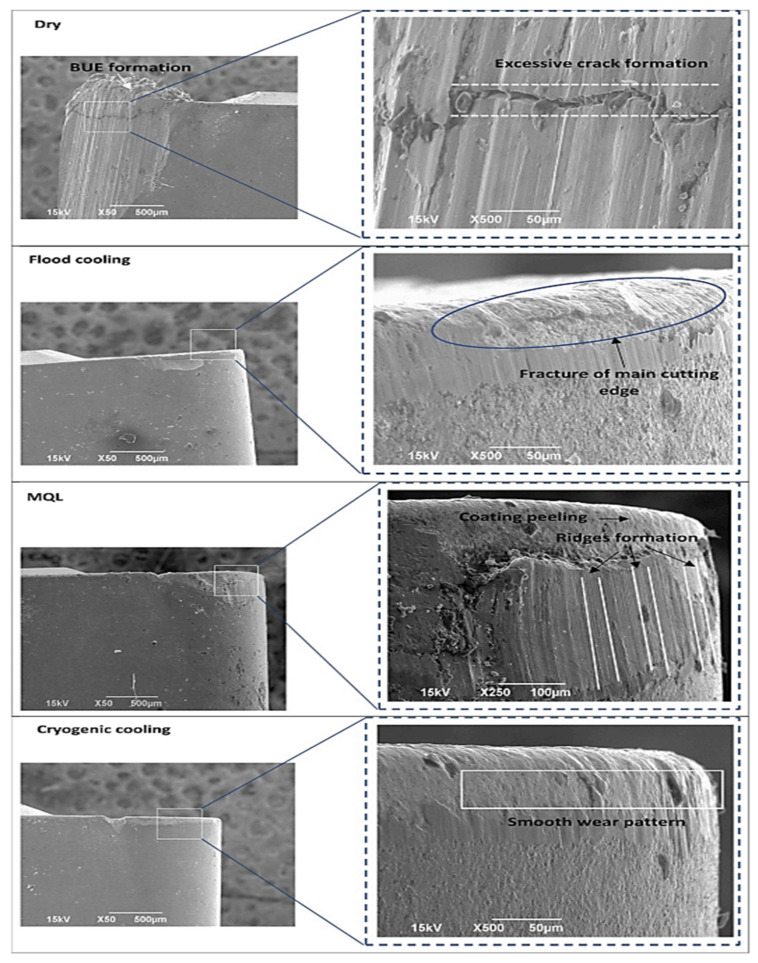
Micrographs of the flank face of cutting inserts under different cutting environments, reprinted with permission from ref. [89]. Copyright 2020 Elsevier Ltd.

The effects of dry, MQL, flood, and cryogenic machining were observed during turning of 15-5 PH SS, and it was noted that cryogenic machining performed well in terms of tool life which was about 44% of flood and 68% of MQL cooling technique. Surface roughness was better in cryogenic and flood cooling as compared to MQL and dry cutting. In Figure 22, SEM images show the smoothness in the wear pattern of the flank face of the tool in cryogenic as compared to other techniques [89].

The growth of global production and the increase of cutting fluids application has caused intensive research concerning economic and environmental aspects of systems for cooling/lubricating the cutting zone. Thus, recently several cooling/lubrication techniques were developed in order to achieve sustainable manufacturing by reducing or eliminating cutting fluids. Currently, the most widely used cooling/lubricating techniques with a low negative effect on the environment and human operator’s health are dry cutting, cryogenic cooling, and minimum quantity lubrication (MQL), etc. [90].

LN2 was found to be good in milling of P20 hardened steel as compared to dry and flood machining. Tool wear was less, about 15%, compared to dry machining, while about 5% compared to wet cooling. Also, it was noted that due to temperature reduction, chip curl was less, which leads to good surface morphology [91]. In the machining of AISI D6 tool steel, a comparison was made between LN2 machining, dry, and wet machining. LN2 was good in surface integrity, and tool life was good, but the production cost for cryogenic setup was more compared to dry machining. This cost varied as the flow rate of LN2 increased [92]. In the milling of AISI D2, the impact of cryogenic cooling was noted compared to a dry and wet cutting environment. Cutting zone temperature was reduced up to 44% by dry and about 36% by wet machining, while cutting forces were reduced by about 40% by dry and about 29% by wet machining [93].

During the study, a comparison was done in the machining of normalized and hardened bearing steel AISI 52100. The response of cryogenic and conventional turning techniques like dry and flood cooling was checked in terms of tool life, surface finish, and productivity. Productivity was higher in cryogenic cooling, and tool life was about 315% in normalized while 15% in the hardened workpiece compared to other techniques. No white layer was formed in cryogenic that are not recommended for machining part. Table 9 shows the MRR for both techniques, and it can be seen that it is about 23% more in cryogenic [94].

**Figure 23:**
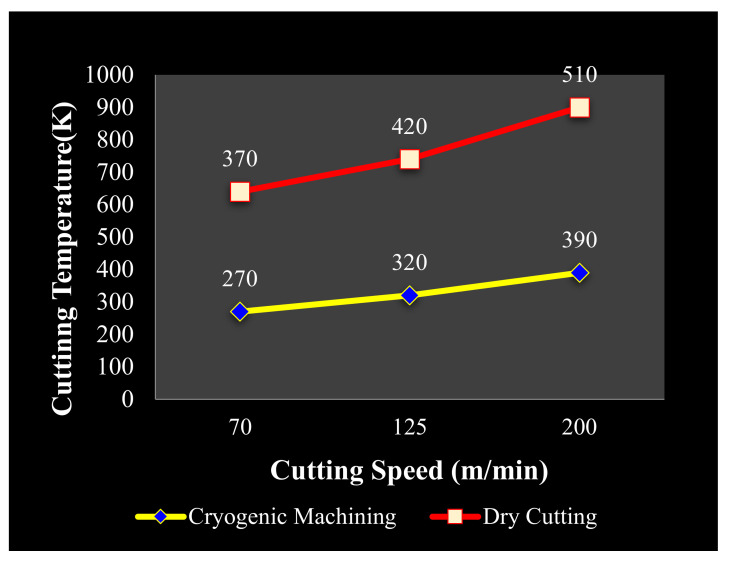
Effect of two cooling techniques on cutting Temperature, reprinted with permission from ref. [95]. Copyright 2018 Elsevier Ltd.

During machining of duplex stainless steel, a comparison was conducted between cryogenic cooling and dry cutting. The tool which was used in the machining was coated carbide. Reduction of cutting zone temperature was observed in the case of cryogenic by 53–58%. Required cutting forces were decreased by 30–43%; also, it was noted that surface finish was improved by 18% to 23%. These results were in comparison with dry cutting. Figure 23 shows the cutting temperature for cryogenic and dry machining, which is less in cryogenic machining [95].

**Figure 24:**
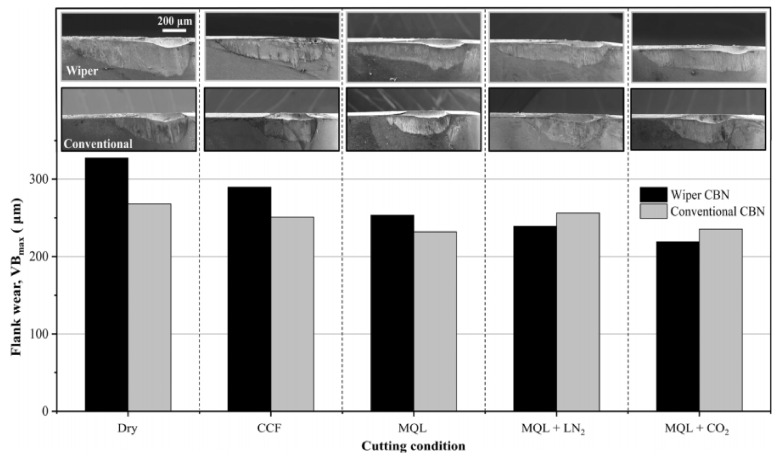
Wear values and SEM images of conventional and wiper CBN inserts under different cutting conditions, reprinted with permission from ref. [96]. Copyright 2020. The Society of Manufacturing Engineers. Published by Elsevier Ltd.

In the hard turning of AISI 52100 bearing steel, the impact of Cryo MQL with two different media (LN_2_ and CO_2_) was evaluated against conventional and dry turning. Machining was done with two different inserts: one was conventional cubic boron nitride (CBN), and the other was a wiper geometry insert. Less flank wear and crater wear were observed using MQL + CO_2_. It was due to the combined effect of minimum quantity lubrication with cryogenic cooling. The surface finish was better, and this technique was found to be eco-friendly. Figure 24 shows the wear pattern of the flank face, which is more in dry cutting, while in Cryo MQL + CO_2_, better wear performance was observed, especially by using the wiper geometry insert [96]. Cryogenic machining has major benefits in the sense of environment and product quality, but some limitations like lack of lubrication and chip cleaning. Also, a drawback is the coldness effect for the operator due to high cooling generation during this process [97].

In a nutshell, the cryogenic cooling technique assists us in minimizing chip adherence on a tool. Its benefits include reduction of wear and tear, increase in tool life, improved surface finish, and a decrease in the coefficient of friction. Although some literature has stated that the cryogenic method is beneficial in all aspects, as mentioned earlier, it has certain limitations, as ascertained by Tushar and Suprabhat in their work [98]. The drawbacks are: (1) cryogenic process demands extra control and monitoring over cooling process, (2) a large amount of machining cost belong to process, so any failure during operational hours lead to high maintenance expenses, (3) liquid nitrogen cannot be reused, (4) it is not acceptable for heat treatment processes, (5) and cryogenic fluid, when operated at low temperature, becomes reactive; therefore, it damages the workpiece by directly contacting it.

### 4.2. Minimum Quantity Lubrication

To avoid using a large amount of cutting fluids, a technique called minimum quantity lubrication, or near dry machining [99], is used in which cutting fluid is supplied at the rate of 100 mL/h. Lawal et al. [100] demonstrated that MQL is a highly competitive approach for a sustainable environment. They explained that minimum usage of cutting fluid in MQL reduces environmental and occupational health hazards. It is well known that metal cutting fluids cause environmental problems. In this case, the amount of cutting oils is greatly reduced which also reduces the environment problem. It was also pointed out that the use of vegetable oils improves the performance of the MQL process, especially in the machining of hard materials, by using water soluble oil in the presence of nano particles. There was no toxic effect generated by using this process which leads to sustainable machining process [101].

**Figure 25:**
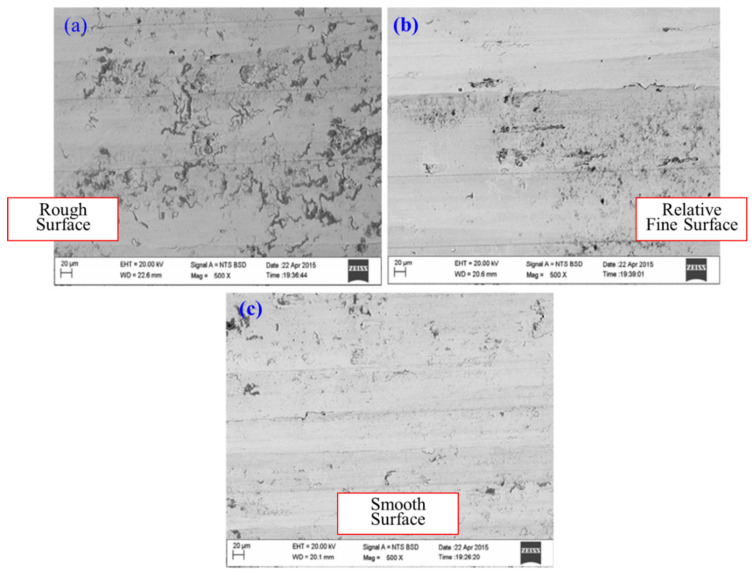
Morphology SEM images; (**a**) By dry turning, (**b**) conventional wet turning, (**c**) and HTMF application, adapted from ref. [102].

Normally, machining is done in dry mode, but the problem which we face is shorter tool life, and sometimes, surface integrity suffers. On the other hand, flooded type coolant application has a higher cost. So, a tradeoff is required in the form of minimal application of lubricants (MQL) which will serve both purposes. In a comparison of wet and MQL, it was found that MQL had better results in tool wear, tool vibration, surface roughness, cutting forces, and cutting temperature during hard turning. About 1.3%, 6.7%, and 8.6% reduction were observed in surface roughness, tool wear, and tool vibration, respectively. Tool wear was less observed in the minimal cutting application as compared to others. Figure 25 shows the surface morphology of three types of cutting techniques in which hard turning with minimal fluid (HTMF) produced the smoothest surface [102].

To avoid cutting fluids, dry cutting can be adopted, but this results in shorter tool life at higher cutting parameters, so near dry machining is recommended. Using cutting oil at optimal speeds serves both economic and environmental [103] purposes. In turning of AISI H13 hardened steel under the MQL method, it was noted that the surface finish was improved. This method also has the benefit of being environmentally friendly due to the minimal use of aerosols and cutting oils [104]. An experiment was conducted on heat-treated AISI 4340 steel with a hardness of 52–54 HRC in MQL and dry turning conditions using different bio-cutting oils. It was observed that surface roughness improved as compared to dry turning. At higher cutting speeds, more than 240 m/min, sudden tool failure was observed under MQL conditions [62].

**Figure 26:**
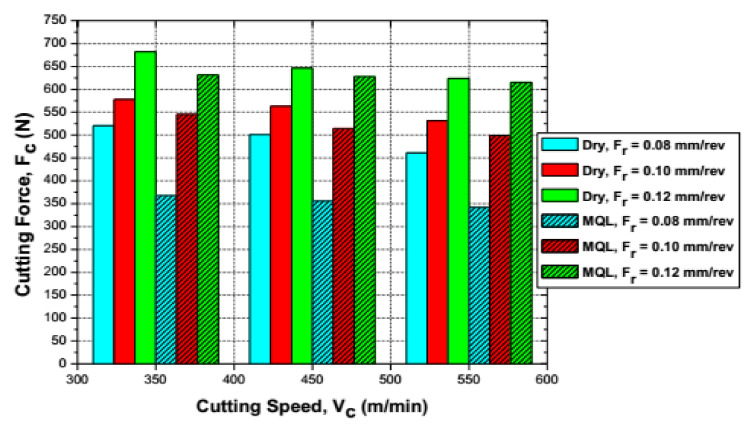
Effect on cutting forces by using Dry and MQL, reprinted with permission from ref. [105]. Copyright 2015 Elsevier B.V.

In the machining of AISI 1045, it was found that the cutting temperature and cutting forces were reduced by 10–30% and 5–28%, respectively, in MQL compared to dry machining (see details in Figure 26). This reduction of temperature leads to better tool life and contributes to sustainable manufacturing [105].

In turning of AISI D2 steel, the effect of the eco-friendly MQL system was observed compared to dry machining in terms of tool life, tool wear, and surface finish. Reduction of about 100 °C was noted in cutting zone temperature, and surface finish was improved up to 91% compared with dry machining. Tool wear was less and tool life was increased about 267% in chemical vapor deposition (CVD) coated tools [106].

In a study [107] to check the sustainability and effectiveness of different cooling and lubrication techniques, it was found that MQL nanofluids and cryogenic were the best techniques in terms of keeping a balance between the sustainable environment and not compromising machinability efficiency.

MQL technique is an efficient process when we compare it with wet machining. About 15% was saved using this technique. It was noted that it has a better effect in the form of a good surface finish and longer tool life compared to dry machining. When we used biodegradable oils, the effectiveness of this technique increased towards the sustainable point of view. Cutting temperature was reduced by about 50%, which reduced the cutting forces as well [108].

Table 10 shows the different cost estimations of different techniques used in machining. MQL was found better in terms of initial setup and tool cost. Cleaning and disposal costs are comparable with other techniques [109].

**Figure 27:**
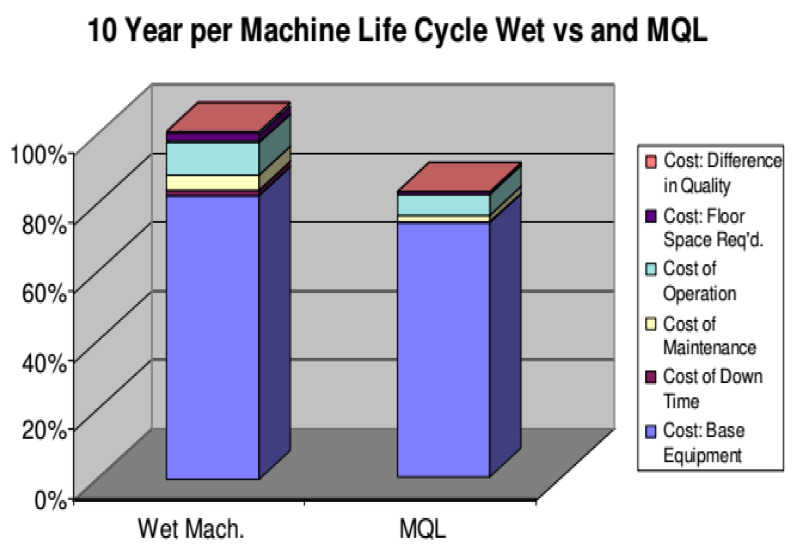
Up to 10 years life cycle of two types of machining techniques, reprinted with permission from ref. [83]. Copyright 2015 Elsevier Ltd.

In an experiment performed on a transmission housing using MQL rather than wet machining, about 15% in savings were achieved. It was noted that due to the reduction of wastewater, it is a sustainable process. One problem is in MQL is the cleaning of chips during machining, especially of hard materials. Figure 27 shows the cost comparison of two types of machining processes, MQL, and wet machining. Operation and maintenance costs are less using MQL. Equipment costs are also less, and the overall cost is about 78% than in wet machining [110].

**Figure 28:**
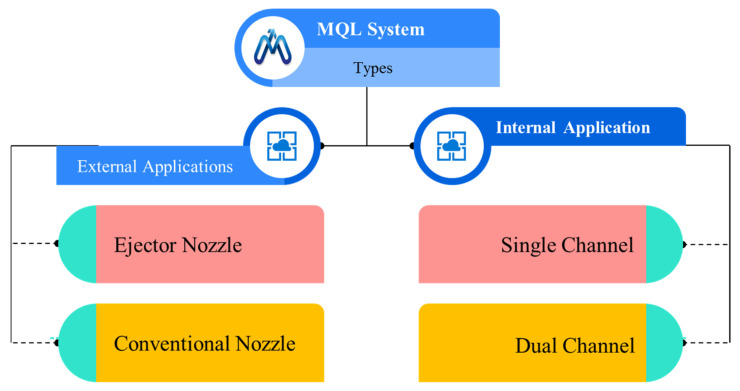
Different types of MQL systems, reprinted from ref. [108]. Copyright 2020 Elsevier Ltd.

MQL can be applied in two types of application methods. Different types of MQL systems are shown in Figure 28. In the external application, a compressed air and oil mixture is fed through an external nozzle to the cutting area from a chamber. There are two types of this system. One has an ejector nozzle in which air and oil are supplied separately to the ejector, and mixing is done after the nozzle. In conventional mixing, it is done before the feeding at the cutting zone. In internal application, the mixture is sent through the spindle and tool to the cutting area of the part [111].

Using cutting fluids at a very large scale in machining creates many environmental problems, so it is necessary to adopt a strategy that minimizes the use of these oils but serves the purpose of machining. Also, governments have imposed restrictions on the disposal of such fluids as these cause damage to natural resources. To avoid environmental, regulatory, and health-related problems, MQL is a better technique that serves most of the purposes and also reduces costs [112].

In the context of industry 4.0, sustainable manufacturing is very important. Research was conducted to check the sustainable aspects of MQL on the machining of difficult-to-cut materials, and it concluded that MQL is a tradeoff between flood type and dry cutting. It has more advantages for the environment and is more cost-effective than other techniques. Skin problems created by metalworking fluid (MWF) were reduced by using MQL [113].

In the machining of a mold of tile industry, the impact of sustainable machining was observed. MQL technique was used for such purpose, and it was noted that by using optimal cutting parameters, a major improvement was achieved in the context of a safe cutting environment. There was an approximate 67% reduction in kg CO_2_, and about 3357 liters of water were saved. Costs were reduced by about 60% [114].

Four types of cooling techniques (dry, MQL, flood, and solid lubricants with compressed air) were investigated in the machining of AISI 1060 in terms of temperature and surface roughness. In all these, MQL was found to be the best from a sustainable point of view. This technique is responsible for lower manufacturing costs and fewer occupational health and safety problems [115]. Due to sustainability, some properties possessed by MQL are high lubricity, high stability and should be biodegradable. Low consumption of oil is very common in these [116,117].

In addition to the above literature, some studies have also been carried out under vegetable oil mixed MQL conditions. Khan et al. [118] machined low alloy steel of grade AISI 9310 using vegetable oil emulsion. They studied the effect of the MQL process on SR, cutting temperature, chip development, and electrode erosion in different cutting environments. They proposed that surface roughness and tool tip wear were extensively reduced under the MQL environment, and flank wear promisingly improved when machining was treated in vegetable oil. Likewise, some investigations are compiled based on conventional machining in MQL conditions. For instance, Braga et al. [119] compared the results of two scenarios; one in MQL state and the second in the mixture of Al-Si (7% Si) alloy. They conducted a drilling process in both conditions and then measured the potency of each. The results yielded the same SR values in both of the aforementioned drilling conditions, which generally confirms the sustainability of vegetable oil-based machining. In another work, Kishawy et al. [120] used Al alloy (Al-356) to examine the effect of high-speed face milling under dry, wet, and MQL setups at various cutting conditions such as speed of cutting up to 5225 m/min. They illustrated that high cutting forces were noted in the case of dry cutting while fewer cutting forces were observed in wet machining. Whereas in MQL, intermediate cutting forces were marked.

MQL has some disadvantages: (1) removal of chips from the machining zone is not carried out properly, (2) MQL permits corrosion in the work parts or in the chips, (3) there must be great care taken in nozzle adjustment, as it should be more than 1 or 2 inches from the tool, (4) MQL is limited to chip heat removal only, it does not cool down the workpiece and tool, (5) mist creation is also one of the major drawbacks of MQL [121].

### 4.3. Dry Cutting

Sustainable manufacturing refers to the use of all available natural resources which reduce environmental pollution. Machining is one of these which is very much energy-intensive, and we have to bring improvements to reduce energy consumption. Using cutting fluids is very common for this process but has tremendous impacts on the environment as there are certain disposal costs associated with it. There is no convenient method to dispose of it after proper treating due to which skin diseases are common. To eliminate these problems, dry machining is used in which the need for cutting oils is eliminated. Dixit et al. [90] reported that the use of dry machining significantly minimized air and water pollution. They called dry cutting an eco-friendly process. Eco-friendly refers to such techniques in which detrimental wastes and tiny particles are excluded. Schultheiss et al. [122] urged that dry machining can be performed without any fluid; therefore, for sustainability, it is more appreciated to engage dry processing than the traditional machining approach where usually dielectric fluid is used to compensate for the generated heat while operating on the work part. Dry cutting is more suitable for low-strength materials, and using coated tools is recommended, which can reduce heat generation [123,124,125,126]. In the machining of 15-5 PHSS hardened steel, an experiment was done to compare different cooling techniques like dry, wet, and cryogenic cooling. Sustainability assessments were conducted, and it was noted that dry cutting is more optimal towards sustainable assessment indicators, but when we talk about the combined effects of productivity and environment, cryogenic machining is good [127].

In the machining of stainless steel under dry cutting conditions, the effect of feed rate, cutting speed, and depth of cut was observed. The main objective was to reduce the energy cost and machining cost, which is the ultimate objective of sustainable machining. It was concluded that at a higher feed rate and cutting speed with a lower depth of cut, the energy consumption was reduced by 33.46% with a 17.81% reduction in machining cost [128]. Dry cutting is more useful in the context of the environment as there is no need to dispose of the water and metalworking cutting fluids. Cost is also saved, but the problem is high cutting zone temperature and shorter tool life if the cutting parameters are high. Surface quality is better than wet cutting [129].

Although dry cutting is good to retain the sustainability factor while machining, it also has certain disadvantages. For example, Chetan et al. [130] and Rotella et al. [77] outlined that adhesion between the electrode and chips takes place in specific tool and workpiece materials. The said issue led to the reduction of the material erosion rate, and thus the quality of the machined surface is compromised. Moreover, there is the chance of a greater heat-affected zone over the surface, which decreases the strength and durability of the workpiece. Therefore, sustainability plays a prime role in the metal processing areas.

### 4.4. Cryogenic Treated Tools

Tool life is very important to increase the productivity of any machining industry. It is necessary to use tools that have a long tool life without the use of cutting oils for environmental protection. What are the requirements for sustainable machining? Cutting tools without any treatment wear very rapidly due to heat generation on the cutting zone. Cryogenic treatment is done on cutting tools to compensate for this. Cryogenic treatment is an add-on process that is required to improve tool life. The ultimate goal is to improve the performance, which cuts down the machining cost. It is a subzero heat treatment process that affects the entire cross-section area of cutting tools. Life enhancement of tools is accomplished by microstructure changes of the tool during cryogenic treatment. Two types of treatments are used; one is shallow, and the other is deep cryogenic treatment. Shallow treatment: −80 °C to −145 °C. Deep cryogenic treatment: −145 °C or below. It was noted that the performance of deep cryogenic treatment is more effective than shallow treatment [131].

Cryogenic treatment is an advanced process for increasing tool life, reducing wear resistance, improving the strength and microstructure of the tool [132,133,134]. With the help of cryogenic treatment on the tool, productivity in terms of tool durability is escalated satisfactorily. Much past literature based on cryogenic treatment has been enlisted. For example, Ramji et al. [135] studied the effect of drilling processes on non-treated and cryogenically treated tools, and a combination of cryogenically treated and heat-treated carbide tipped drills on thrust, SR, and torque of drilled holes in diverse cutting conditions. They concluded that cutting forces, thrust, and torque were reduced when cryogenic treated and a combination of heat-treated carbide insert was used. Gill et al. [136] evaluated the effect of cryogenic treatment of tools on cooling rate. They demonstrated that when cooling and heating are performed at different rates (say 0.5 °C/min and 1 °C/min), then the wear resistance of the tool and micro-cracks on the surface was improved, respectively. Another study conducted by Silva et al. [74] reported the impact of cryogenic treatment of M2 HSS tools and said that 65–34.3% improvement was observed in the reduction of tool fracture while drilling on steel. Cryogenic treatment has numerous benefits in traditional machining, including milling, drilling, and turning. It has been extensively used outside the conventional machining zone for microstructure analysis and wear resistance tests for increasing tool life [136,137,138,139,140].

Furthermore, an experiment was done to check the impact of cryogenic treatment on Tungsten carbide inserts. It was noted that the inserts’ life was increased up to 36% with deep cryogenic treatment compared to non-treated inserts. Cutting forces were lesser, and performance was more consistent. Tool life was about 56% in deep cryogenic treatment than by non-treated insert at cutting speed of 110 m/min [92,93].

Cryogenic treatment has many benefits due to its enhancement of cutting tool properties by changing the austenite phase to the marten site phase by heat treatment. By doing this, the hardness and toughness of cutting tools improved [141,142,143]. In the machining of PHSS, cryogenic treat inserts were used. Due to lesser flank wear, tool life was improved as compared to non-treated tools. These tools resulted in lesser cutting forces, enhanced surface finish with longer tool life [144]. In the machining of 15-5 PHSS cryo- treated inserts were used, and it was noted that cutting forces were reduced, and due to high hardness and strength, the wear of the tool was less as compared to conventional types of tools [145].

Deep cryogenic treatment in hard turning of AISI D2 steel with ceramic cutting tools improves the surface roughness by 32.97%, and improvement in tool life was observed 21.79% [146]. In the turning of C 45 steel, the impact of cryo-treated tungsten carbide inserts was noted compared to non-treated inserts. Treated inserts were found best in machinability and long tool life. Tool tip temperature was decreased due to higher thermal conductivity by cryogenic treatment. This treatment is limited to smooth turning [147]. Contrarily, in cryogenic treatment of cutting tools, machinability increases, and due to good thermal conductivity, cutting temperature decreased. These types of tools are not preferable for interrupted cutting due to breakage problems. This statement indirectly limits the use of cryogenic treatment of tools.

### 4.5. Solid Lubricants

In the solid lubricant-assisted machining of hardened steel, it was found that this technique is suitable for an ecofriendly environment with less cost of production and helps in the reduction of waste as well as occupational health and safety. It was noted that as demand for sustainable machining is increasing day by day, so solid lubricant assisted machining is emerging as a sustainable alternative machining process [148].

It was noted in a review that the performance of solid lubricants at higher cutting parameters is high, which leads to enhanced productivity. Also, it was observed that there is no negative impact while using these, but the issue is selecting the right type of solid lubricant [136].

In the turning of hardened steel, the effect of solid lubricants was noted, and it was discovered that Molybdenum disulfide is better than graphite. It was observed that solid lubricants are better than dry or wet turning in terms of improved surface finish and from an environmental point of view. The good lubricating effect of these solid lubricants caused the reduction of cutting zone temperature and tool wear. This is becoming a good alternative to dry and wet turning [149].

In the turning of AISI 1040 steel, the impact of solid lubricants (MoS_2_) was noted in terms of toxic effect, surface finish, and machinability efficiency. It was concluded that the surface finish was improved by 5% to 30%. The chip thickness ratio was reduced. The friction was reduced in this process, so the material removal rate was high, which leads to high productivity. Also, not using cutting fluids leads to better environmental impact [150].

**Figure 29:**
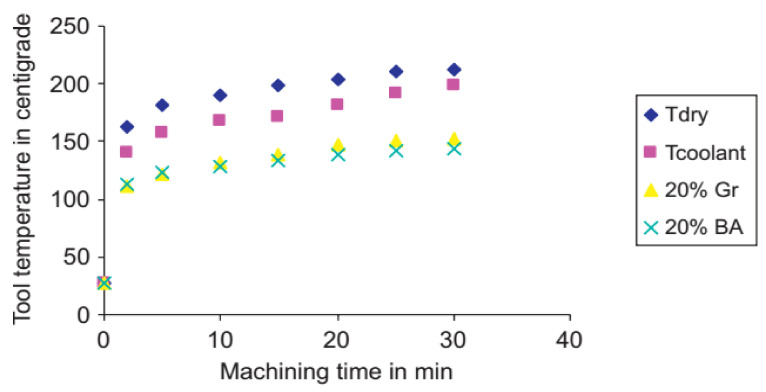
Tool temperature with time in different cutting techniques, reprinted with permission from ref. [151]. Copyright 2008 Elsevier Ltd.

In machining, the effect of SAE 40 oil with different percentages of graphite and boric acid was studied. It resulted that the boric acid (20%) in SAE 40 oil was performing well. The surface finish was improved, and less tool wear and lesser cutting forces were observed to boric acid lubricious film formation, which lessens the friction forces and cutting temperature. Figure 29 shows the impact of boric acid and graphite on cutting temperature compared to dry and wet cooling. Boric acid and graphite were comparable, and with the passage of cutting time, the performance of Boric acid fond good [151]. Graphite was used in grinding, and it was found that it had numerous effects on the process. The major difference was in the surface finish of the workpiece as in other conventional cutting oils, which were very much improved [152].

In the machining of AISI 1040, the effect of nanoparticles in cutting fluid was noted, and it was found that thermal conductivity increased, and heat transfer rate increased about 6%, which increased tool life. It was found that about 1% addition of nanoparticles in cutting fluids is optimal [153].

**Figure 30:**
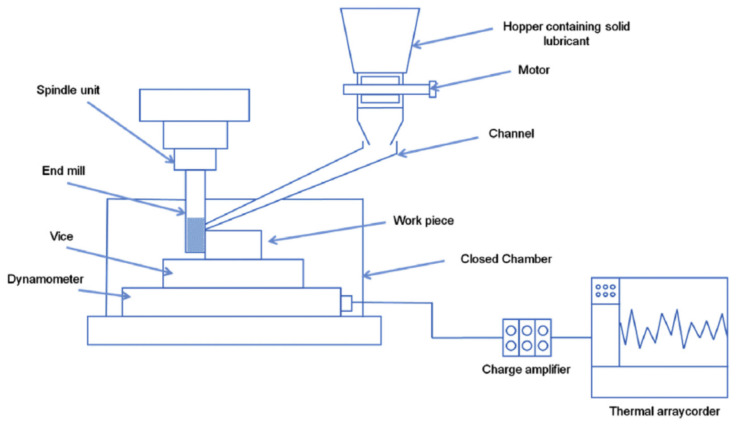
Lubricant feeding system, reprinted with permission from ref. [157]. Copyright 2005 Elsevier Ltd.

Different types of solid lubricants like MoS_2_, CuO, SiO_2_, and CaF_2_, etc., are useful due to the low strength of bonding between these shears off rapidly. They are also nontoxic and produce a good lubricity effect [154]. In the turning of bearing steel, the effect of Cu nano-fluid with vegetable oil under minimum quantity lubrication was noted. It was found that surface roughness was improved by about 51% due to self-laminated film formation between the tool and workpiece, which reduced the friction. Due to the better thermal conductivity of Cu nanofluid, a reduction in cutting zone temperature was observed, about 21%, compared to vegetable oil machining [155]. Solid lubricant-assisted machining is an ecofriendly technique that contributes to improving the economical aspect of any industry. Improved tool life and higher productivity were observed in the machining of AISI 304 steel. Surface roughness was improved up to 39%, which was improved due to less wear of the tool tip [156]. All lubricants were supplied to the machining area with the help of a special feeding system, as shown in Figure 30 [157]. Solid lubricants have several drawbacks over other sustainable techniques such as (i) high wear rate with a high coefficient of friction, (ii) some lubricants have poor heat dissipation due to low thermal conductivity, like polymers lubricants, (iii) comprises poor self-absorption of heat ability which disturbs the durability of lubricants [158].

### 4.6. Alternative Cutting Fluids

The use of cutting oils/lubricants causes diseases in employees. To minimize the effect of these oils, some user-friendly oils like vegetable-based oils and other bio-degradable oils can be used. The usage of these oils improved the surface finish and enhanced the tool life. Due to less coefficient of friction than other mineral oils, the machining efficiency improved, and cutting forces were reduced. These are less toxic than other mineral oils, etc. Table 11 shows the positive and negative impacts of vegetable-based oils on energy, cost, and environment. These are efficient in terms of all these parameters like in enhancement of tool life, less requirement of energy due to reduction in forces and eco-friendly. However, there are some negative issues like fume generation and cleaning problems, as chips adhere to oil [159].

In experimental machining of AISI 304, two types of vegetable-based cutting oils were used. One was sunflower oil, and the other was canola oil. The comparison was made with the semi-synthetic mineral oil. It was noted that the above two oils performed well in the context of being environmentally friendly and in cost reduction. The surface finish was also improved. It was noted that the performance of canola oil with the additive was best in the overall scenario [160].

Soybean and sunflower oils were tested as metalworking fluids, and it was noted that these had a good impact on the environment and were suitable for cutting and forming operations. These are the best alternative to cutting oils [161]. Table 12 shows the different advantages and disadvantages of vegetable oils. They are cost-efficient and less toxic than mineral oils. The low rate of environmental pollution and high biodegradability make these safer for use. One drawback is low thermal stability [162,163,164,165,166,167,168,169,170].

About 95% usage of vegetable-based oil in Brazil was reported in contrast to petroleum-based oils due to their biodegradable properties and ability to be extracted from natural resources. Soybean oil is the most commonly used in industrial applications [171]. In addition to their positive impact on the environment, it was reported that surface roughness was improved by 31.6% when vegetable oils were used in MQL [100].

In the turning of alloy steel ASIS 9310, vegetable oil performed excellently in terms of the material removal rate, which lead to high productivity. Machining performance was increased by using these oils by 117% in terms of tool life, and thrust forces were also reduced. Also, it has a less negative environmental impact [7]. Vegetable oils have a high boiling point and molecular weight, due to which the chances of vaporization are less than other neat oils. Less smoke is produced, so it is less hazardous for the working environment as well as for people. The product quality is improved by the effect of the lubricating film. Friction and heat generation were lower [118]. In the turning of AISI 4340 stainless steel, three different oils, palm oil, sunflower oil, and coconut oil, were used. Sunflower oil performed well in terms of surface finish and chip compression ratio. One drawback of vegetable oils is the generation of smoke due to a lower flash point [172].

### 4.7. Air/Gas/Vapor Cooling

**Figure 31:**
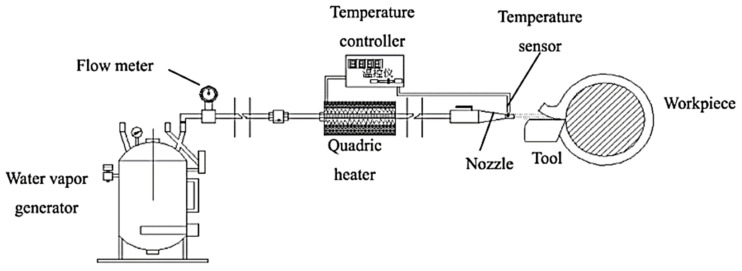
Vapor generator device and feeding system, reprinted with permission from ref. [173]. Copyright 2004 Elsevier Ltd.

The use of cutting fluids causes environmental damage and health-related issues. In order to avoid these issues, a green cutting environment is being created. In this environment, the use of water vapor plays a major role because there is no need for recycling or disposal, and it is non-toxic and environmentally friendly. The setup diagram is below in Figure 31.

**Figure 32:**
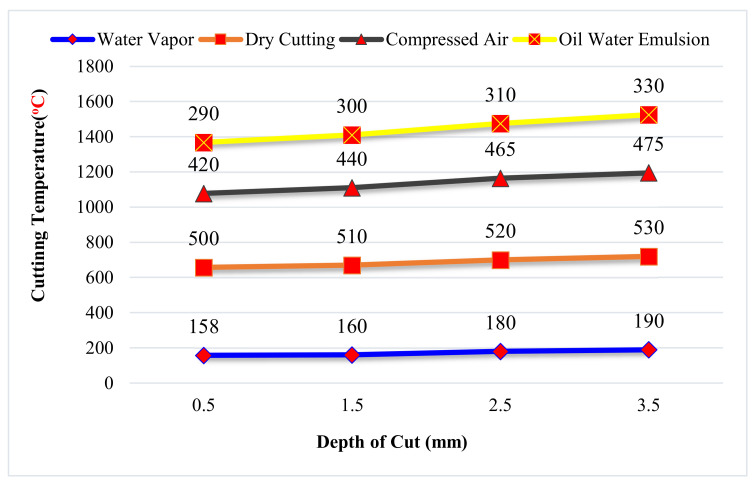
Cutting Temperature Variation on different Depth of cut, reprinted with permission from ref. [173]. Copyright 2004 Elsevier Ltd.

Temperature reduction, cutting force reduction and improvement in the surface finish is a positive impact of this technology. Below, Figure 32 shows the temperature comparison between different modes of cutting lubrication techniques in which the use of water vapor is the best technique compared to dry cutting, compressed air, and oil-water emulsion [173].

Cold air cooling is best during machining as it mitigates the environmental and health issues caused due to use of coolants. Energy consumption increased by 20%, but coolant cost was reduced by 80%, which is an economically good impact [174]. In the literature, different gases have been exploited as a coolant for sustainable machining of steel, i.e., carbon dioxide (CO_2_), argon, water vapor, oxygen, and nitrogen, as depicted by Kim et al. [175] and Yamazaki et al. [176] in their investigations. Contrarily, it comprises some drawbacks; for example, rough turning is not appropriate for the gas/air cooling method. It also acquires an additional setup for the supplement of gas particles to the machining area. As a coolant, compressed air is not suitable for machining a superalloy like Inconel alloy. From an environmental perspective, CO_2_ as a gas is not compatible for greenhouse effect.

The past studies warrant the use of air or gas as a coolant to sustain the process environmentally. Liu et al. [177] performed machining on ANSI 1045 steel against a P10 carbide tool under different concentrations of gases and oils. For instance, water vapors (WV), a mixture of CO_2_ and O_2_, a combination of WV and CO_2_, a grouping of WV and O_2_, dry machining, and wet machining under oil-H_2_O emulsion were prepared for processing. They deduced that cutting forces improved significantly with increased tool life up to 4 to 5 times and 2 to 3 times with CO_2_ state and WV, respectively. Junyan et al. [178] collated the two different machining contexts; process under WV and state of dry machining. They evaluated the impact of the K20 carbide insert on the performance of ANSI 304 stainless steel in the aforementioned two machining situations. They extrapolated that better results were obtained with WV followed by dry machining in terms of improvement in tool life, a reduction in cutting forces of 25 to 30%, and modification in surface integrity.

**Figure 33:**
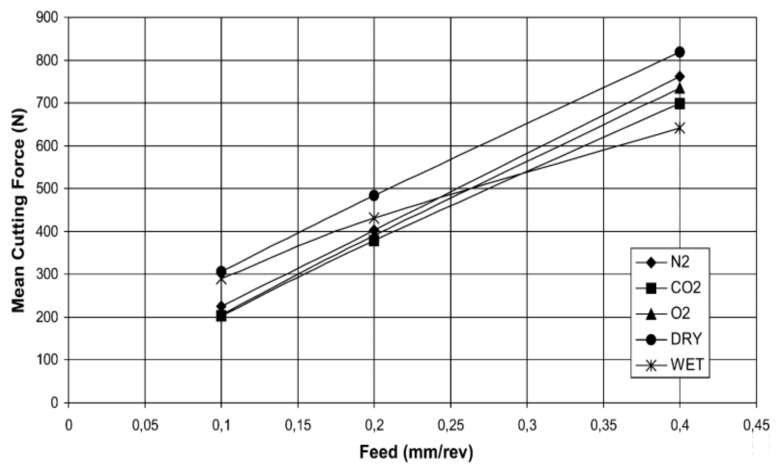
Variation in cutting force by using different machining techniques, reprinted with permission from ref. [179]. Copyright 2004 Elsevier B.V.

In the machining of AISI 1040, the comparison was carried out between gases applications, wet and dry machining. Three gases were taken, oxygen, nitrogen, and carbon dioxide. It was found that gas application had better result in surface quality, cutting zone temperature, and cutting forces, etc. CO_2_ had a better cooling effect than other gases used, and the cutting forces and thrust forces were less using this gas compared to other gases. At lower feed, good surface quality was achieved with gas compared to wet machining, in which surface quality improved at a high feed. Figure 33 shows the relation of mean cutting force with feed in dry, wet, and different gases. CO_2_ was best in all other techniques [179].

### 4.8. High-Pressure Coolant (HPC)

**Figure 34:**
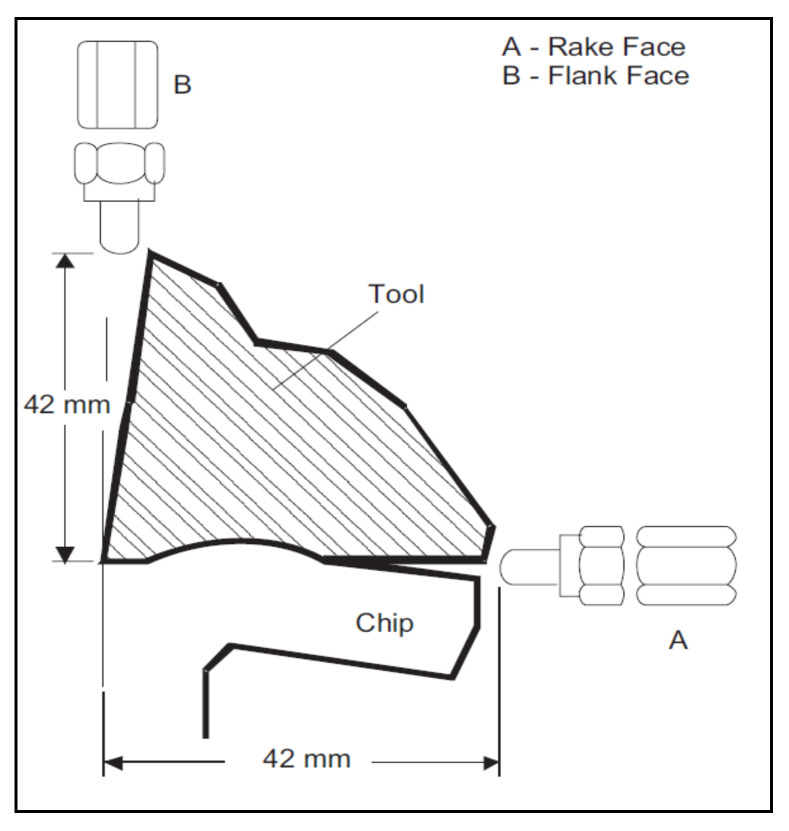
Position of the tool with respect to the workpiece, reprinted with permission from ref. [182]. Copyright 2006 Elsevier Ltd.

This is another widely accepted technique in the manufacturing industry. Conventional machining mostly uses one mechanical mechanism, but HPC is usually comprised of three systems: mechanical, thermal, and tribological controls, which makes it impressive and valuable in high-speed machining [180]. High-speed machining is useful in the following conditions, (i) difficult to machine materials, (ii) high speed and feed, (iii) deep-hole drilling, (iv) continuous chips production [181]. HPC generally provides high pressure to the coolant, which allows the deep flow of the fluid between the work-electrode spaces or contact regions of tools and chips, as specified in Figure 34 [182]. The effect of the above phenomenon improves tool life, decreases the consumption of cutting fluid, and maintains the temperature of the work part [183]. It has been found from the literature that HPC not only offers less TWR but also gives superior cooling properties, which results in lessened contact distance as the force of coolant pressure lifts the chip away from tool faces [184]. Ezugwu et al. [185] investigated that boron nitride (BN) and ceramic tools are not fit for high-speed processing of Ti-alloys with HPC supply because it begins the nose rupture and generates discontinuous chips which damage the cutting edges.

It was mentioned earlier that an increase in coolant supply with greater pressure increases the tool life. A study confirmed that tool life is raised by 740% when pressure and coolant speed are set at 203 bar and 50 m/min, respectively. In addition, chip formation is also affected by varying the cutting conditions and coolant pressure at acceptable levels [186]. Kumar et al. [187] have evaluated the effect of HPC on the machining performance of ASSAB 718 steel. The improvement in tool wear, flank wear, chip shape and thickness, and cutting forces are governed by HPC. Dhar et al. [188] assessed the consequence of HPC on chips, tool life, and roundness deviation while drilling of AISI 4340 steel. They investigated the outcomes under HPC drilling with the dry drilling process. The results summarized that small chip thickness, less roundness, and minimal tool wear were observed via HPC drilling. Thus, researchers called it a more beneficial process than drilling under conventional coolant. Naves et al. [189] presented the machinability of AISI 316 austenitic steel by employing HPC. They used 5% and 10% vegetable oil with coolant at different ranges of pressure (100, 150, 200 bar) against carbide tool inserts. They concluded that flank wear was significantly lower when pressure up to 100 bars with a 10% concentration of fluid was applied. The above literature successfully showed that HPC is a highly effective method for achieving long tool life, minimum chip size, and, most importantly, the consumption of fluid is decreased by 50%.

## 5. Discussion

**Figure 35:**
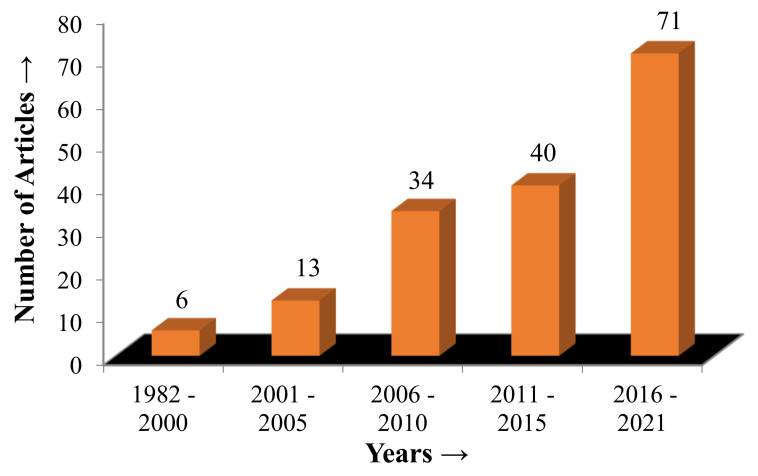
Graphical representation of the number of cited articles in different year bands.

This section broadly investigates the sustainability aspect of steel governed by different machining techniques, such as expressed in Figure 10. To build a state-of-the-art review, comprehensive literature has been studied regarding the sustainability point of view for the manufacture of steel. For this reason, the key letters and strings were treated to reveal the different studies relevant to the above-mentioned case for the literature survey. The literature survey comprises published work obtained from various sources of Journals, including Science Direct, Emerald, Springer, and other publishers. The last 25–30 years articles, 43% from the past five years, were cited in this study as presented in the graph shown in Figure 35.

It has been regarded that steel is the most popular material and is preferred widely in diverse application sectors, i.e., automotive, aerospace, and manufacturing industries, etc. However, advancement in manufacturing areas, together with the requirement of cleaner production, demands sustainable machining techniques. Therefore, this review article is contributing towards the sustainable methods needed for the machining of steel. In addition to the above discussion, this study is also presented as a guide for the selection of the best technique that gives net zero-emission in their manufacturing products. The important published work corresponds to sustainable techniques employed for the machining of steel is described below.

Numerous researchers claimed that MQL is the most suitable technique for achieving a sustainable machining environment followed by conventional processes such as drilling, milling, and grinding, etc. [190,191,192,193]. Najiha et al. [194] said that MQL is considered a cleaner production process due to its cost-effectiveness and ensuring the safety of both workers and the environment. This statement is also validated by other researchers; for instance, Boswell et al. [111] and Eltaggaz et al. [195] predicted that MQL consumed a minimum quantity of cutting liquid which directly reduces the emission of hazardous fumes, and thus the performance of MQL process was upgraded. Moreover, vegetable oil and non-natural esters are the most commonly used fluids in MQL owing to superb biodegradability and non-toxicity, as stated by Boswell et al. in their study [111]. Dhar and Khan [196] explained that some benefits of the aforementioned fluids over conventional metalworking lubricants are:

i. They provide high MRR with a small cutting time;
ii. The small electrode erosion rate;
iii. They are good absorbers at high pressure;
iv. Minimal vaporization and evaporation lead to being environmentally sustainable.

Synthetic ester, sometimes also known as vegetable oil, is also a promising fluid in order to sustain the machining process due to its high boiling point, excellent flashpoint, and low viscosity, as implied by Dixit et al. [117] in their investigation. Hence, both stated fluids extensively used in the MQL process are the best alternatives in terms of suitability than other conventional liquids.

Cryogenic is another fundamental sustainable approach used for the cutting of steel by manipulating cryogenic fluid at optimum temperature. To keep the cutting temperature low, a coolant like nitrogen gas is used because of its non-corrosive and non-combustible nature. Pereira et al. [67] carried out turning operation on AISI 304 material and inferred that a 50% improvement in tool life with a 30% reduction in cutting speed was commemorated under cryogenic machining conditions. Pusavec et al. [197] evaluated the impact of cryogenic machining on Inconel 718 alloy by taking surface integrity as a responses parameter. They have used various mixtures of cryogenic liquids and found good surface asperities over the machined region with a cryogenic cutting procedure. Another study by Pusavec et al. [198] was written about the effect of various machining applications (such as cryogenic cooling, MQL, dry machining, cryo-lubricant machining) on the same alloy of Ni (Inconel 718) by constructing a response surface methodology (RSM) model. They validated the model with the support of an ANOVA study. The results were explicated that cryogenic cutting fluid or lubrication significantly improved the performance of machining while treating. Machai and Biermann [199] tested the tool life (TL) during machining of Ti-1023 at specific cutting conditions (Cutting rate = 50–150 m/min, Feed = 0.1 mm, machined depth = 0.3 mm, and stroke length = 50–250 m) under wet and CO_2_ blend. They summarized that TL is raised approximately by two times with the cryogenic machining as compared to wet operating conditions. Machining under emulsion state also generated large size craters than that of cryogenic condition.

Cutting lubricants improve the design attributes of the machining, but they are strictly avoided by some researchers due to the production of health-hazardous fumes and gases during processing which causes serious diseases for the workers. Researchers have practiced the machining operation without any fluid referred to as “dry machining (DM)”. Many manufacturing companies, especially those which produce metallic products, are adopting this technology owing to the freedom from environmental impacts. However, DM has certain drawbacks, which prevent its usage at a level as high as the rest of the techniques which used cutting fluids. Gyanendra and Prabir [129] enlisted some disadvantages of DM, which are mentioned below:

i. Excessively raise the temperature of the cutting zone, which results in poor TL;
ii. Heat affected zones are enlarged, which tends to decrease the strength of the specimen;
iii. Surface finish compromised at such elevated conditions;
iv. Geometric accuracy and dimensional accuracy of the work part is significantly altered;
v. The DM has a challenge to machine the difficult-to-cut materials;
vi. In comparison to other sustainable machining techniques, DM has high costs and less productivity.

There are numerous studies based on DM. For example, Servaraj et al. [200] assessed the effect of cutting speed (80–120 m/min) and feed rate (0.04–0.12 mm/rev) on cutting forces at a DOC of 0.5 mm under DM state. They have tested all experiments on the stainless steel (SS) material. They proposed that magnitude of cutting forces is significantly remodeled with a small alteration in feed rate. Fernandez-Abia et al. [201] also performed experimentation under DM environment at a cutting speed of 37–870 m/min, feed rate of 0.2 mm/rev, and 0.1 mm DOC while turning AISI 303 SS. They have taken two responses viz cutting capabilities and chips geometry. They deduced that a cutting rate greater than 450 m/min yielded minimal cutting forces along with satisfactorily decreased chip thickness. Salem and Ahmad [202] optimized the design parameters, such that surface integrity and power consumption, of 316 SS under DM situation using Boron Nitride (BN) electrode. An RSM methodology was developed to validate the machining parameters. The results pointed toward the reduction in power consumption up to 6.8%, with an increase in the surface finish of about 13.9%.

Another prime technique used to ascertain the sustainability perspective of steel can be accomplished with the help of using various cutting fluids. Many pieces of research claimed the amplification of machining performance in terms of output responses like cutting rate, MRR, TWR, and SR when different cutting fluids in the dielectric are exercised. For instance, Kashif et al. [203] comprehensively examined the dispersion of graphene nano-powder mixed in the dielectric medium onto the output parameters (i.e., MRR & TWR) of electric discharge machining (EDM) using three electrodes (copper, brass, and aluminum). They proposed that graphene particles in the dielectric disperse the sparking by raising the plasma channel, which leads to raise the MRR and reduce the TWR. Although there are numerous cutting fluids, vegetable oil-based dielectric combinations are mostly preferred owing to being environmentally friendly, renewable, non-toxic, non-hazardous, and high biodegradability [125]. Vegetable oil also possesses a high freezing point, good corrosion resistance, and is thermally considered stable [204]. Cetin et al. [205] conducted a study to illustrate the impact of canola oil, sunflower oil, and mineral oils on the machining performance of an AISI 304L work part by noticing the SR, cutting forces, and feed forces. They suggested that both vegetable oils provide better surface asperities over the machined surface. Other than this, vegetable oil is outperformed in terms of less feed and cutting forces, followed by mineral oil. Hence, different powder mixed dielectric influences the machining processes and improved the results.

Table 13 shows the comparison in terms of some response parameters like surface finish and MRR, cutting temperature, tool life, and cutting forces. Tool life was best in cryogenic cooling and Cryo treatment of tools. Dry cutting was not found comparable with other techniques in terms of all these parameters, but from an environmental point of view, it was good. The surface finish was best in cryogenic cooling and solid lubricants technique. The material removal rate was observed to be greater in the solid lubricants technique.

After careful review of the presented literature, it has been inferred that sustainable techniques are environmental-friendly and manifest as non-hazardous for human beings. If the demand of manufacturers is to achieve a high MRR with a good surface finish, then the use of solid lubricants will be preferred. While, if the need is limited to high tool life with excellent surface asperities on the machined part, then cryogenic cooling will be favored. Similarly, sometimes studies are only restricted to minimum cutting forces, then dry machining would be used irrespective of the other machining attributes. The different challenges possessed by the sustainable techniques while their implementation is presented in the subsequent section. Then the paper is summarized in the conclusion section with the discussion of future directions.

## 6. Challenges in Sustainable Machining

Different challenges faced for the implementation of sustainable techniques are following.

### 6.1. Lack of Awareness Regarding These Techniques

Most industries are not aware of these latest techniques, which can contribute considerably in terms of productivity improvement and a green and safe environment for workers and surroundings. They only trust conventional methods and consider these a technical requirement. Top management should be equipped with the latest knowledge of the world’s reforms in the field of machining of parts.

### 6.2. Lack of Management Commitment

For the implementation of these techniques in any manufacturing industry, a change mindset is most important. Management should be willing to provide all needed resources for effectiveness, but in most industries, this commitment is not present.

### 6.3. High Equipment Cost

No doubt these techniques are very helpful and of paramount importance to the industries for today and tomorrow, but some techniques like cryogenic machining need much attention because right now, their setup cost is high. Indeed, most industries do not know the payback of these, which makes them reluctant to implement.

### 6.4. Usage of Old Technology

In most industries, old and conventional machines are being used for the manufacturing of parts. There is very little or absence of provision for installation of equipment for working of these techniques. For installation, a huge cost is required for the replacement of the existing system.

### 6.5. Fear of Losing Business during Adoption of These Techniques

It is a myth in conventional industries that whenever they try a new system, it will lead them towards loss of production. It seems that there is not enough time for the trial of any new technology, which is the wrong concept. Without taking a risk, no improvement will occur.

## 7. Post-Processing Challenges of Additive Manufactured Steel

Additive manufactured steel parts are widely used in different engineering applications. However, geometric inaccuracy and poor surface integrity disallow the use of steel components after their manufacturing through additive manufacturing techniques. From this perspective, post-processing operations are performed to mitigate the above issues. Those post-processes include drilling, milling, grinding, blasting, and tapping, etc. The challenges inherent with the aforesaid traditional processes have been explained by the National Institute of Standards and Technology (NIST), as far as additive manufactured (AM) steel is concerned.

The residual, tensile (outer surface), and compressive (inner surface) stresses still remain in the steel component after its production from AM, which hinder the use of such parts of steel via milling, drilling, and other conventional processes because it can be generated trust forces, vibrations, and high frequency that may decrease the tool life rapidly, as discussed by Brandon and Eric in their study [206]. They also reported that residual stress also altered the chips’ formation owing to high tensile forces at the outer surface when plastic deformation is reached.

The fabrication of steel parts via direct energy deposition (DED) has inhomogeneities because of inappropriate cooling and porosity that tend to affect the surface finish of the desired steel parts when turning operation is carried out at certain input variables. Teo et al. [207] also studied the effect of the post-processing technique (sandblasting) on the AM 316L stainless steel part. The surface quality issue governed by the DED process has been successfully eliminated by the sandblasting process, but the introduction of surface damage and peel-off layer takes place. They reported that the peel-off layer could be removed either by electro-polishing technique or by adding abrasive particles; however, it leads to another problem of corrosion. Therefore, steel parts comprising limitations towards machining after their fabrication through AM process.

## 8. Conclusions

Sustainable machining techniques became the need of the hour to fulfill environmental regulations and to improve operator’s safety. These techniques play an important role in any industry in terms of economic, social, and environmental benefits. To maximize productivity and to make products market competitive, sustainable machining techniques should be adopted. The following main points are concluded for understanding and implementation:

- Cryogenic machining is a very popular technique in terms of its excellent cooling impact, which leads to longer tool life and good surface integrity. It does not require residual cleaning as in conventional cutting, but some drawbacks are still there like, chips cleaning problem and frostbite hazard associated with the operator’s health.
- Dry machining is good in terms of an environmental point of view but leads to low surface integrity of the product and higher tooling cost.
- MQL technique is an intermittent solution between dry and cryogenic in terms of machining cost and product quality. However, problem is chip evacuation is a problem.
- In cryogenic treatment of cutting tools, machinability increases, and due to good thermal conductivity, the cutting temperature decreases. This type of tool is not suitable for interrupted cutting due to breakage problems.
- Solid lubricants are found more effective in terms of surface integrity of parts, and tool life increases as the heat transfer rate increased.
- Vegetable oils are found to be good from an environmental point of view as there is no need to dispose of these as compared to mineral oils. Due to low flash points, smoke is produced during the turning of steel. Sometimes cleaning may be problematic as chips engaged with oil which is difficult to separate.
- Air-gas cooling is a good technique for the environment compared to conventional coolants, but energy costs increased about 20%.

Numerous studies have been conducted on the sustainability of steel in various application conditions. As steel is emerging mostly in automotive sectors, along with other large setups like aerospace, nuclear power plant, marine areas, and biomedical equipment, etc. Organizations are conscious of environmental problems, which ultimately put the life of humans at risk. Therefore, it is much more necessary to examine the sustainability point of discussion on steel material. This review was compiled to investigate the sustainable machining techniques for the steel material. Further, the details presented in this review can act as a guide in selecting the best solution to be integrated towards achieving net zero-emission in their manufacturing products.

## 9. Future Implications

This review highlights the major limitations like frostbite hazard in cryogenic machining and initial setup cost, which is difficult to afford by any local industry. A comprehensive investigation is required to mitigate the aforesaid issue of cryogenic machining by ensuring a controlled temperature environment. The mathematical modeling of the sustainable cutting mechanisms with respect to the cutting of steel is still an area that needs special attention.

## Figures and Tables

**Table 1:** Chemical composition of HSLA Steel, reprinted with permission from ref. [39]. Copyright 2018 Elsevier Ltd.

Elements	Cu	Ni	Cr	Mn	Si	Mo	C	Nb	S	P
wt. %	1.25	0.83	0.78	0.54	0.37	0.19	0.07	0.03	0.024	0.022

**Table 2:** Mechanical characteristics of HSLA steel, reprinted with permission from ref. [39]. Copyright 2018 Elsevier Ltd.

Properties	Units	Values
Yield Stress	(MPa)	450 ± 32
Ultimate strength	(MPa)	778 ± 17
Elastic Modulus	(GPa)	203 ± 5
Total Strain	%	21 ± 2

**Table 3:** Mechanical features of low alloy steels (grade 11 & 22), reprinted with permission from ref. [41]. Copyright 2007 Elsevier B.V.

Alloys	Yield Stress (MPa)	Ultimate Tensile Stress (MPa)	Elongation in %
1CrMoV	205	415	30
2.25Cr1Mo	205	415	30

**Table 4:** Chemical composition of different grades of low alloy steels, reprinted with permission from refs. [41,42]. Copyright 2015 Elsevier Ltd.

Alloys	C	Si	Mn	P	S	Ni	Fe
Fe-0.1C-1.5Mn	0.10	0.01	1.48	0.001	0.002	0.01	Balance
Fe-0.1C-3Mn	0.10	0.02	2.96	0.001	0.002	0.01	Balance
Fe-0.1C-1.5Ni	0.09	0.01	0.02	0.001	0.002	1.58	Balance
Fe-0.1C-3Ni	0.09	0.01	0.02	0.001	0.002	3.16	Balance
1CrMoV	0.15	0.50	0.60	0.025	0.025	0.03	Balance
2.25Cr1Mo	0.082	0.23	0.41	0.051	0.0054	0.03	Balance

**Table 5:** Elemental composition of some martensitic alloys, reprinted with permission from refs. [43,44,45]. Copyright 2020 International Atomic Energy Agency (IAEA).

Alloys	Cr	Mo	V	Nb	C	Mn	Cu	Si	N	Ni	P	S	W
9Cr-1Mo	8.55	0.88	0.21	0.08	0.1	0.51	0.18	0.32	0.035	0.15	0.012	0.005	-
9Cr-1MoVNb	8.44	0.89	0.24	0.08	0.086	0.37	0.03	0.16	0.054	0.11	0.012	0.003	-
9Cr-1MoVNb-2Ni	8.57	0.98	0.22	0.066	0.064	-	-	-	0.053	2.17	-	-	0.01
12Cr-1MoVW	11.99	0.93	0.27	0.018	0.21	-	-	-	0.020	0.43	-	-	0.54

**Table 6:** Mechanical characteristics of martensitic alloys, reprinted with permission from refs. [43,44,45]. Copyright 2020 International Atomic Energy Agency (IAEA).

Alloys	Yield Strength (MPa)	Ultimate Tensile Stress (MPa)	Elongation in %
9Cr-1Mo	533	683	26.0
9Cr-1MoVNb	547	697	11.9
9Cr-1MoVNb-2Ni	148	171	19.1
12Cr-1MoVW	110	142	19.6

**Table 7:** Elemental composition of some austenitic alloys, reprinted with permission from refs. [36,46]. Copyright 2018 Elsevier Ltd.

Alloys	Fe	C	Mn	Si	Mo	Co	Cr	Cu	Ni	Others
ASS304L	70.78	0.025	1.140	0.410	0.360	0.210	18.40	0.180	8.190	0.305
ASS316L	67.69	0.018	1.28	0.38	2.42	0.21	16.63	0.21	10.85	0.312
AISI 304	Balance	0.06	3.97	0.49	0.008	0.11	17.61	1.17	8.85	0.076
AISI 201	Balance	0.04	7.38	0.588	0.008	0.072	17.40	2.17	3.13	0.292

**Table 8:** Chemical composition of some tool steel alloys, reprinted with permission from ref. [48]. Copyright 2018 MDPI Metals.

Alloys	C	Si	Mn	P	S	Ni	Cr	Mo	Cu	V	W
AISI D2	1.56	0.24	0.25	0.025	0.001	0.175	11.31	0.83	0.14	0.25	-
AISI M4	1.33	0.33	0.26	0.03	0.03	0.3	4.25	4.88	0.25	4.12	5.88
HWS	1.08	1.38	0.34	-	-	-	7.80	1.86	-	2.66	1.73

**Table 9:** Optimum productivity comparison through MRR, reprinted with permission from ref. [94]. Copyright 2012 Elsevier B.V.

Material	Conventional Turing (mm^3^/min)	Cryogenic Turning (mm^3^/min)
Normalized AISI 52100	61,600 (Flood)	75,600
Hardened AISI 52100	5606 (Dry)	6300

**Table 10:** Qualitative Cost Estimation data for different cooling/lubricating techniques, reprinted with permission from ref. [109]. Copyright 2017 Elsevier Ltd. The symbols used to depict Very low (*), Low (**), Medium (***), High (****), Very High (*****).

Sr. No.	Type	Raw Material Cost	Fluid Consumption	Tool Cost	Equipment Costs	Cleaning Costs	Disposal Cost
1	Cutting fluid	**	*****	**	****	*****	*****
2	Dry Machining	*	*	*****	*	*	***
3	MQL	**	**	**	***	**	**
4	Cryogenic Cooling	***	***	***	*****	*	*
5	Gaseous Cooling	***	***	*****	****	*	*
6	Sustainable Cutting fluid	***	****	**	****	****	***
7	Solid lubricant	****	***	***	***	***	****
8	Nanofluids	*****	****	***	****	****	*****

**Table 11:** A critical analysis of environmental aspects in bio-degradable oil aided machining, reprinted with permission from ref. [159]. Copyright 2019 Elsevier Ltd.

Influence	Performance Issues	Energy	Cost	Environment
Positive	Improved Tool Wear profile	Lower specific cutting energy due to reduced force	Improve performance of bio-based oils reduce overall cost	Eco-friendly cooling lubricating agent
	Increased Tool life	Reduced temperature	Cost of recycling	Recycling of oils can be done
	improved surface finish and less friction	Energy consumption during production of bio-oils	Cost of bio-oil coolant sometimes higher than conventional coolant	Fluids from chips need to be separated before chip processing
Negative	Conventional application mode lacks penetration	Mode of oil application determines the additional energy consumption	Cost of additives if used	MQL spray cause inhalation problem
	Adhere with chips-separation of oils from chips are required	-	-	Fumes can cause problems to human

**Table 12:** Different Advantages and disadvantages of vegetable oils used as lubricants.

Advantages	Disadvantages
High Biodegradability	Poor Corrosion Protection
Less environmental pollution	Low Thermal Stability
Low Volatility	High Freezing Points
Lesser production cost	Oxidative Stability
High Flash Points	
Low Toxicity	
High Viscosity indices	
Wide Production Possibilities	
Compatibility with other additives	

**Table 13:** Comparison between different sustainable Techniques. (× Bad ×× Good ××× Better ×××× Best).

Technique	Tool Life	Surface Finish	MRR	Cutting Temperature	Cutting Forces
Cryogenic Cooling	××××	××××	×××	××××	×××
MQL	×××	×××	××	×××	×××
Dry Cutting	×	××	××	×	××
Solid Lubricants	×××	××××	××××	××××	×××
Air/Vapor/Gas	××	××	×××	×××	×××
Cryogenic treatment	××××	×××	××	×××	××××
Alternative cutting fluids	×××	×××	×××	×××	×××

## Data Availability

The raw/processed data required to reproduce these findings cannot be shared at this time as the data also forms part of an ongoing study.

## Nomenclature

This section describes the nomenclature of various abbreviations used in this study:

MQL	Minimum Quantity Lubrication
CEO	Chief executive officer
OEM	Original Equipment Manufacturers
LAS	Low Alloy Steel
HAS	High Alloy Steel
LNG	Liquefied Natural Gas
AISC	American Institute of Steel Construction
HSLA	High Strength Low Alloy
MPa	Mega Pascal
GPa	Giga Pascal
SS	Structural Steel
HRS	Heat Resistant Steels
AISI	American Iron and Steel Institute
PRISMA	Preferred Reporting Items for Systematic Reviews and Meta-Analysis
SR	Surface Roughness
MRR	Material Removal Rate
TWR	Tool Wear Rate
CBN	Cubic Boron Nitride
HTMF	Hard Turning with Minimal Fluid
SEM	Scanning Electron Microscope
HRC	Hardness Rockwell C Scale
CVD	Chemical Vapour Deposition
MWF	Metalworking Fluid
MoS_2_	Molybdenum disulfide
DOC	Depth of Cut
DM	Dry Machining
TL	Tool Life
EDM	Electric Discharge Machining
LOF	Lack-of-fusion
